# CXCR5 and TLR4 signals synergistically enhance non‐small cell lung cancer progression

**DOI:** 10.1002/ctm2.1547

**Published:** 2024-01-18

**Authors:** Ji Hye Shin, Mi‐Jeong Kim, Ji Young Kim, Yeeun Kang, Duk‐Hwan Kim, Soo‐Kyung Jeong, Eunyoung Chun, Ki‐Young Lee

**Affiliations:** ^1^ Department of Immunology and Samsung Biomedical Research Institute Sungkyunkwan University School of Medicine Suwon Gyeonggi‐do Republic of Korea; ^2^ Department of Molecular Cell Biology Sungkyunkwan University School of Medicine Suwon Gyeonggi‐do Republic of Korea; ^3^ R&D Center CHA Vaccine Institute Seongnam‐si Gyeonggi‐do Republic of Korea; ^4^ Department of Health Science and Technology, Samsung Advanced Institute for Health Science and Technology, Sungkyunkwan University School of Medicine Samsung Medical Center, 81 Irwon‐ro, Gangnam‐gu Seoul Republic of Korea

Dear Editor,

Chemokines and their receptors play essential roles in neoplastic transformation, tumour cell growth and survival, and organ‐specific metastasis during carcinogenesism.^1^, ^2^, ^3^ Of various CXC chemokines, CXCL13 and its related receptor CXCR5 have been implicated in lung cancer progression.^2^ However, the molecular and cellular mechanisms by which the CXCR5–CXCL13 signal axis is functionally regulated in lung cancer progression are still poorly understood. Through clinical microarray data analysis of primary non‐small cell lung cancer (NSCLC; *n* = 42) patients, we found that the up‐regulation of CXCR5 and CXCL13 or CXCR5 and TLR4 in lung tumour tissues versus matched lung normal tissues was significantly associated with gene sets related to cancer module, lung fibrosis, VEGF, chemokine, cytokine and TLR signalling pathway. Through functional analysis with *CXCR5*‐knockout (*CXCR5*‐KO) human lung cancer cells generated by CRISPR/Cas9 gene editing method, we found that the CXCR5–CXCL13 axis was functionally linked to TLR4 signalling through activation of NF‐κB for lung cancer progression, strongly suggesting that our clinically comparative results and functional investigations of TLR4–CXCR5 signalling network in lung cancer could potentially contribute to translational approaches for the development of lung cancer therapeutic agents.

Gene expression profiling interactive analysis (http://gepia.cancer‐pku.cn/detail.php?gene = CXCR5) revealed a positive correlation between CXCR5 and CXCL13 expression in lung adenocarcinoma (LUAD) (Figure S1A; *p* = 3.8e−08, *R* = 0.25). The expression of CXCL13 was significantly enhanced in LUAD and lung squamous cell carcinoma (Figures S1B and C). To clinically get insight into the role of the CXCR5–CXCL13 axis, we utilised microarray data of primary NSCLC patients’ lung tumour tissues (*n* = 42; Table S1) and their matched lung normal tissues (*n* = 42). We performed a gene set enrichment analysis (GSEA; https://www.gsea‐msigdb.org/gsea/index.jsp) to identify biological processes and pathways associated with the expression of CXCR5 and CXCL13. By differential magnitudes (△Mag) of CXCR5 and CXCL13 expression between lung tumour tissues and matched lung normal tissues, we sorted and selected 21 LTTs for the GSEA (Figure 1A, 14 CXCR5^up^CXCL13^up^ LTTs and seven CXCR5^down^CXCL13^down^ LTTs; Table S2). GSEA results revealed that 13 cancer module gene sets were significantly enriched in CXCR5^up^CXCL13^up^ LTTs versus CXCR5^down^CXCL13^down^ LTTs (Figures 1B–G and S2A–G). Moreover, gene sets related to lung fibrosis, VEGF, chemokine signalling, cytokine and JAK–STAT signalling pathways were highly enriched in CXCR5^up^CXCL13^up^ LTTs compared with those in CXCR5^down^CXCL13^down^ LTTs (Figures 1H–L), suggesting that expression levels of CXCR5 and CXCL13 might be associated with lung cancer. To functionally verify the role of CXCR5, *CXCR5*‐KO A549 and H1299 lung cancer cells were generated using CRISPR/Cas9 gene‐editing method (Figures 1M and N, *CXCR5*‐KO A549; Figure 1O, *CXCR5*‐KO H1299).^4^, ^5^ Wound healing assay and transwell assay to evaluate cancer cell migration, cell proliferation assay and anchorage‐dependent or ‐independent colony formation assay were performed using control (Ctrl) and *CXCR5*‐KO lung cancer cells treated with or without CXCL13. Cancer cell migration was significantly induced in Ctrl A549 and Ctrl H1299 cells treated with CXCL13, whereas it was markedly attenuated in *CXCR5*‐KO A549 and *CXCR5*‐KO H1299 cells (Figures 2A–D, wound healing assay; Figures 2E–H, transwell assay). Upon CXCL13 stimulation, cell proliferation and anchorage‐dependent colony formation ability were markedly attenuated in *CXCR5*‐KO A549 or *CXCR5*‐KO H1299 cells treated with CXCL13 as compared to those in Ctrl A549 or Ctrl H1299 cells treated with CXCL13 (Figures 2I–J, cell proliferation; Figures 2K–N, anchorage‐dependent colony formation). Similar results were observed in anchorage‐independent colony formation assay (Figures S3A–D). Taken together, these results suggest that the CXCR5–CXCL13 signalling axis is functionally implicated in lung cancer progression.

**FIGURE 1:**
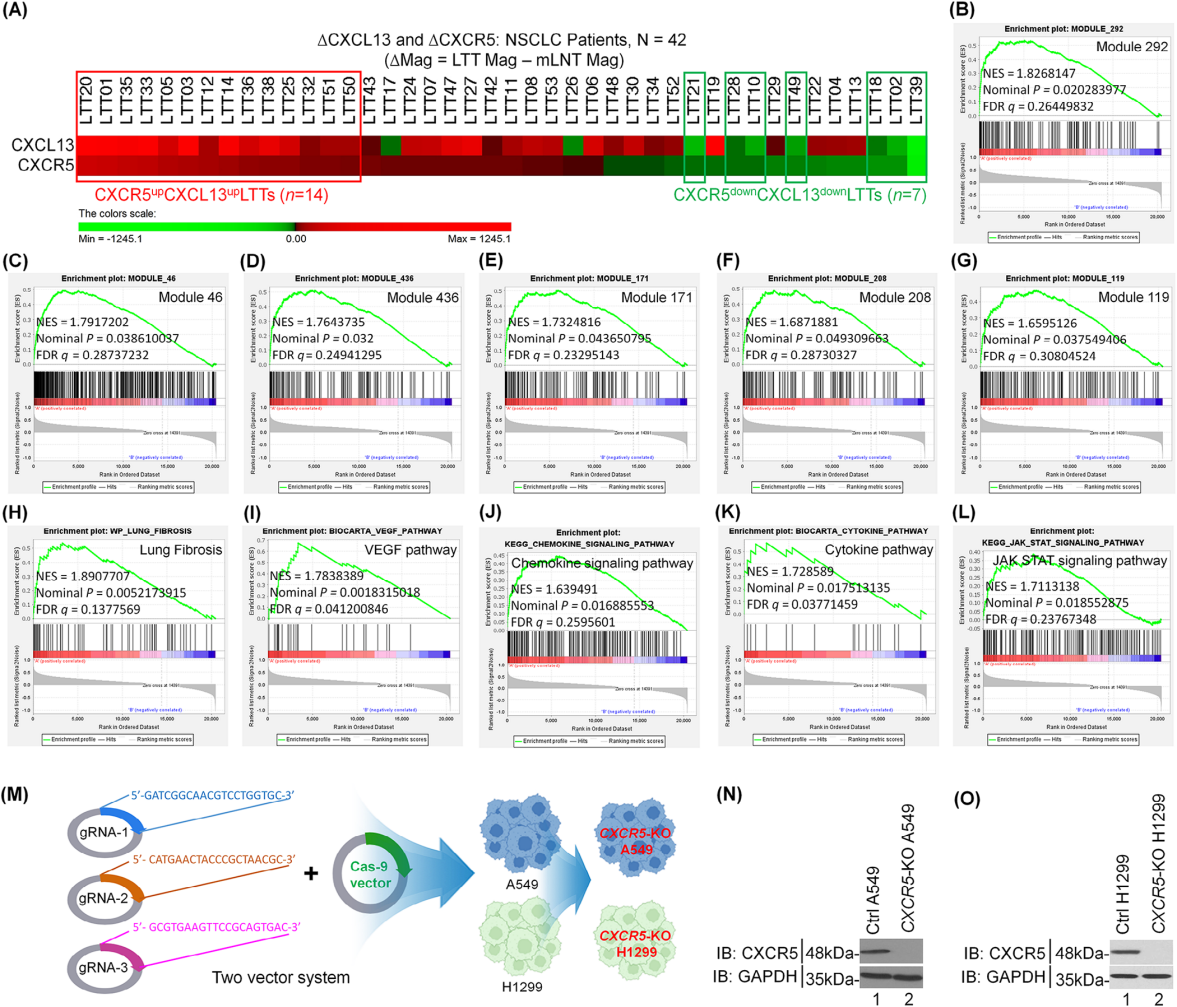
Expression levels of CXCR5 and CXCL13 are up‐regulated in NSCLC patients. (A) Forty‐two lung tumour tissues of NSCLC patients are listed according to different magnitudes (∆Mag) of ∆CXCR5 and ∆CXCL13 expression in lung tumour tissues (LTTs) versus matched lung normal tissues (mLNTs). Fourteen CXCR5^up^CXCL13^up^ LTTs (red boxes) and seven CXCR5^down^CXCL13^down^ LTTs (green boxes) were selected for GSEA. The colour scale indicates ∆Mag value. (B–G) GSEA was performed for 14 CXCR5^up^CXCL13^up^ LTTs versus seven CXCR5^down^CXCL13^down^ LTTs. Gene sets for six cancer modules are presented. NES, nominal *p* value and FDR *q*‐values are indicated in the inner panel. (H–L) Gene sets for lung fibrosis (H), VEGF pathway (I), chemokine signalling pathway (J), cytokine pathway (K) and JAK–STAT signalling pathway (L) are presented. NES, nominal *p* value and FDR *q* values are indicated in the inner panel. (M–O) Generation of *CXCR5*‐knockout (*CXCR5*‐KO) lung cancer cells using two vector systems of CRISPR/Cas9 gene‐editing method. Three *CXCR5*‐guide RNA sequences for CRISPR/Cas9 were designed as indicated. gRNA vector expressing gRNA of CXCR5 and Cas9 vector expressing Cas9 were transfected into A549 cells and H1299 cells (M). After two weeks, colonies were isolated from 96‐well plates. Expression levels of CXCR5 in A549 cells (N) and H1299 cells (O) were analysed with western blotting.

**FIGURE 2:**
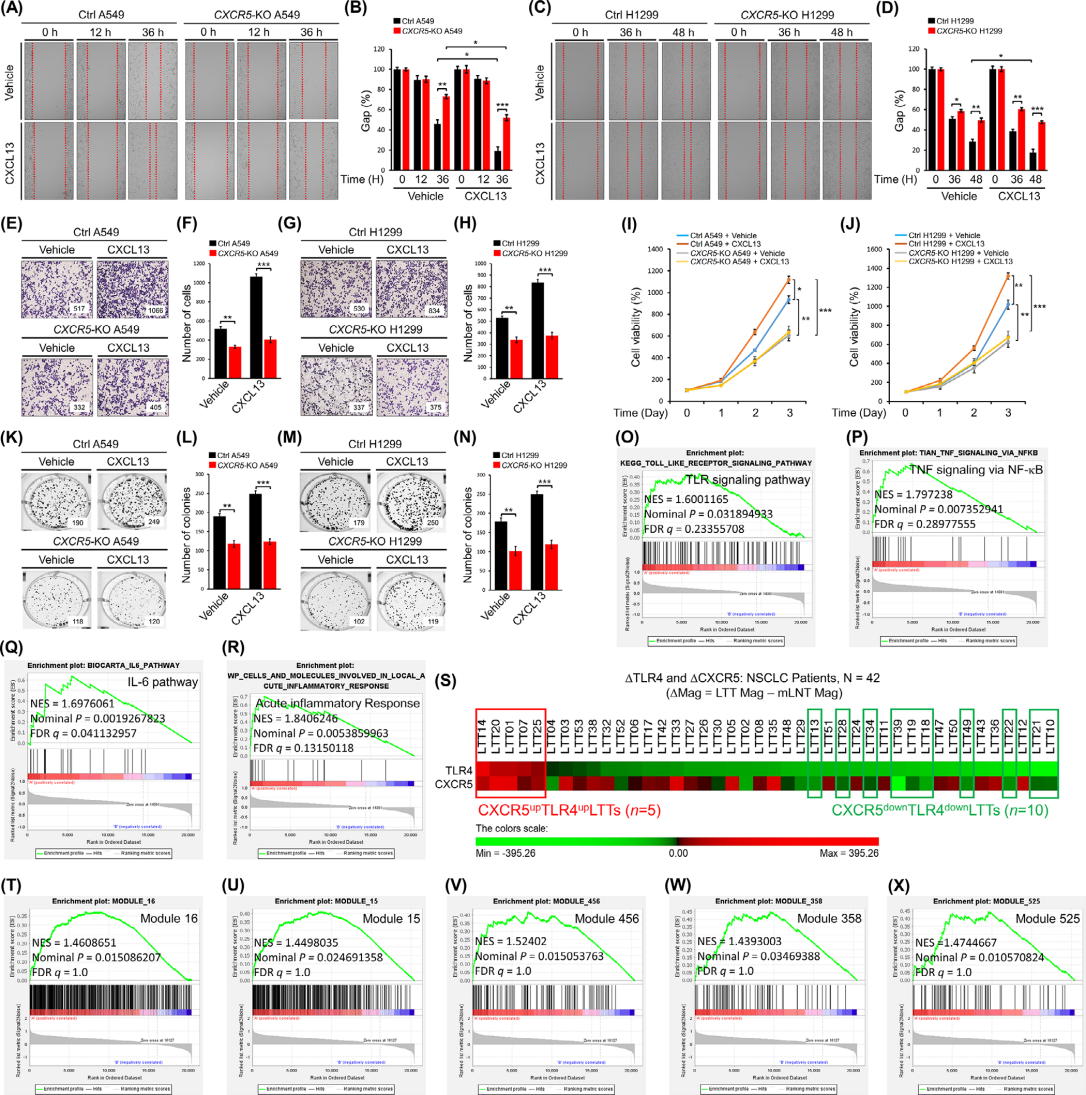
*CXCR5*‐KO lung cancer cells attenuate cell migration, proliferation and colony formation in response to CXCL13. (A–D). Ctrl A549 and *CXCR5*‐KO A549 cells (A and B) or Ctrl H1299 and *CXCR5*‐KO H1299 cells (C and D) were treated with vehicle (DMSO, 0.1% v/v concentration) and CXCL13 (40 ng/mL) for different time periods as indicated. The residual gap between migrating cells from the opposing wound edge is expressed as a percentage of the initial scraped area (±SD, *n* = 3 different plates) (B and D). (E–H) Ctrl A549 and *CXCR5*‐KO A549 cells (E and F) or Ctrl H1299 and *CXCR5*‐KO H1299 cells (G and H) were treated with vehicle (DMSO, 0.1% v/v concentration) and CXCL13 (30 ng/mL) for 24 h. The number of migrating cells was counted. Results are presented as mean ± SD of three independent experiments (F and H). (I and J). Ctrl A549 and *CXCR5*‐KO A549 cells (I) or Ctrl H1299 and *CXCR5*‐KO H1299 cells (J) were treated with vehicle (DMSO, 0.1% v/v concentration) and CXCL13 (10 ng/mL) for different time periods as indicated. MTT assay was then performed as described in *Material and Methods*. Results are presented as mean ± SD of three independent experiments. (K–N) Ctrl A549 and *CXCR5*‐KO A549 cells (K and L) or Ctrl H1299 and *CXCR5*‐KO H1299 cells (M and N) were treated with vehicle (DMSO, 0.1% v/v concentration) and CXCL13 (30 ng/mL) for 8 days. The number of colonies was counted. Results are presented as mean ± SD of three independent experiments (L and N). (O–R) GSEA was performed for 14 CXCR5^up^CXCL13^up^ LTTs versus seven CXCR5^down^CXCL13^down^ LTTs (Figure 1A). Gene sets for TLR signalling pathway (O), TNF‐signalling via NF‐κB (P), IL‐6 pathway (Q) and local acute inflammatory response (R) are presented. NES, nominal *p* value and FDR *q* values are indicated in the inner panel. (S) Forty‐two lung tumour tissues of NSCLC patients were listed according to different magnitudes (∆Mag) of ∆TLR4 and ∆CXCR5 expression in lung tumour tissues (LTTs) versus matched lung normal tissues (mLNTs). Five CXCR5^up^TLR4^up^ LTTs (red boxes) and 10 CXCR5^down^TLR4^down^ LTTs (green boxes) were selected for GSEA. The colour scale indicates ∆Mag value. (T–X) GSEA was performed for five CXCR5^up^TLR4^up^ LTTs versus 10 CXCR5^down^TLR4^down^ LTTs. Gene sets for five cancer modules are presented. NES, nominal *p* value and FDR *q* values are indicated in the inner panel. *, *p* < .05; **, *p* < .01; and, ***, *p* < .001.

Previous studies have shown that TLRs, such as TLR2 and TLR4, contribute to cell proliferation by modulating the CXCR5–CXCL13 signalling axis and regulate lung cancer progression.^6^, ^7^, ^8^ Importantly, *CXCR5*‐knockdown cells exhibit attenuation of the activation of NF‐κB induced by a TLR4 agonist LPS,^9^ suggesting that the CXCR5 signal might be functionally associated with the TLR4 signal through NF‐κB activation. Interestingly, we found that gene sets related to innate responses, such as TLR signalling pathway, TNF‐signalling via NF‐κB, IL‐6 pathway, local acute inflammatory response, cytokine–cytokine receptor interaction, CXCR3 pathway, AP1 pathway and chemokine receptors bind chemokines, were significantly enriched in CXCR5^up^CXCL13^up^ LTTs versus CXCR5^down^CXCL13^down^ LTTs (Figures 2O–R and S4A–D). To get insight into the association between TLR4 and CXCR5 in lung cancer, we further selected five CXCR5^up^TLR4^up^ LTTs and 10 CXCR5^down^TLR4^down^ LTTs in 42 NSCLCs (Figure 2S and Table S2) and performed GSEA. Ten gene sets related to cancer modules were highly enriched in five CXCR5^up^TLR4^up^ LTTs versus 10 CXCR5^down^TLR4^down^ LTTs (Figures 2T–X and S5A–E). Additionally, gene sets related to the TLR signalling pathway, cytosolic DNA sensing pathway, NOD‐like receptor signalling pathway, RIG‐I‐like receptor signalling pathway, and complement cascade pathway were significantly enriched in five CXCR5^up^TLR4^up^ LTTs (Figures 3A and S6A–D). To explore the functional effect between TLR4 and CXCR5 signals, we performed biochemical studies. Upon TLR4 stimulation with LPS, the expression of CXCR5 was significantly increased in A549 cells (Figure 3B, lane 2−4 vs. lane 1). Importantly, phosphorylation levels of IKKs and p65 were increased in A549 cells treated with LPS or CXCL13. They were markedly elevated in response to co‐treatment of LPS and CXCL13 (Figures 3C–E). Consistently, NF‐κB activity and production levels of IL‐6 and IL‐1β cytokines were significantly elevated in the group co‐treated with LPS and CXCL13 (Figure 3F, NF‐κB activity; Figure 3G, IL‐6; Figure 3H, IL‐1β). To determine whether the activation of NF‐κB in *CXCR5*‐KO lung cancer cells was affected, Ctrl A549, Ctrl H1299, *CXCR5*‐KO A549 and *CXCR5*‐KO H1299 cells were treated with LPS, CXCL13 or LPS plus CXCL13. Phosphorylation levels of IKKs and p65 were significantly attenuated in *CXCR5*‐KO A549 and *CXCR5*‐KO H1299 cells treated with LPS, CXCL13 or LPS plus CXCL13, as compared to those in Ctrl A549 and Ctrl H1299 cells (Figure 3I, Ctrl A549 and *CXCR5*‐KO A549; Figure S7, Ctrl H1299 and *CXCR5*‐KO H1299). Consistent results were observed in the NF‐κB reporter assay (Figure S8A, Ctrl A549 and *CXCR5*‐KO A549; Figure S8B, Ctrl H1299 and *CXCR5*‐KO H1299). Moreover, the phosphorylation of AKT, which is involved in cell proliferation and survival, was markedly attenuated in *CXCR5*‐KO A549 and *CXCR5*‐KO H1299 cells treated with LPS, CXCL13 or LPS plus CXCL13, as compared with those in Ctrl A549 and Ctrl H1299 cells (Pho‐AKT; Figures 3I and S7). These results suggest that CXCR5 and TLR4 signals can synergistically induce the activation of NF‐κB and AKT for proliferation and survival (Figure 3J), thereby regulating lung cancer growth. Given the above results, we examined whether CXCR5 and TLR4 signals regulated lung cancer progression. Ctrl A549, Ctrl H1299, *CXCR5*‐KO A549 and *CXCR5*‐KO H1299 cells were treated with CXCL13, LPS or CXCL13 plus LPS. Wound healing assay and transwell migration assay revealed that *CXCR5*‐KO A549 and *CXCR5*‐KO H1299 cells showed reduced migration ability in response to CXCL13, LPS or CXCL13 plus LPS compared with Ctrl A549 and Ctrl H1299 cells (Figures 4A‐H, *CXCR5*‐KO A549 and *CXCR5*‐KO H1299 vs. Ctrl A549 and Ctrl H1299). Moreover, *CXCR5*‐KO A549 and *CXCR5*‐KO H1299 cells treated with CXCL13, LPS or CXCL13 plus LPS showed significantly attenuated proliferation ability (Figure 4I, Ctrl A549 and *CXCR5*‐KO A549; Figure 4J, Ctrl H1299 and *CXCR5*‐KO H1299). Consistently, Ctrl A549 and Ctrl H1299 cells treated with CXCL13, LPS or CXCL13 plus LPS showed significantly enhanced anchorage‐dependent and ‐independent colony formation ability, whereas *CXCR5*‐KO A549 and *CXCR5*‐KO H1299 cells showed marked attenuation of colony formation ability (Figures 4K and L, Ctrl A549 and *CXCR5*‐KO A549; Figures 4M and N, Ctrl H1299 and *CXCR5*‐KO H1299; Figures S9A and B, Ctrl A549 and *CXCR5*‐KO A549; Figures S9C and D, Ctrl H1299 and *CXCR5*‐KO H1299). We finally assessed whether the deficiency of CXCR5 is affected on tumour spheroid formation. We performed the 3D tumour spheroid assay with Ctrl A549 or *CXCR5*‐KO A549 cells treated with vehicle, CXCL13, LPS or CXCL13 plus LPS. The spheroid size was increased in Ctrl A549 cells treated with CXCL13, LPS or CXCL13 plus LPS, as compared with those treated with vehicle (Figures 4O and P, Ctrl A549 treated with CXCL13, LPS or CXCL13 plus LPS vs. vehicle). Importantly, the spheroid size was significantly decreased in *CXCR5*‐KO A549 cells treated with vehicle, CXCL13, LPS or CXCL13 plus LPS, as compared with those of Ctrl A549 cells (Figures 4O and P, *CXCR5*‐KO A549 vs. Ctrl A549).

**FIGURE 3:**
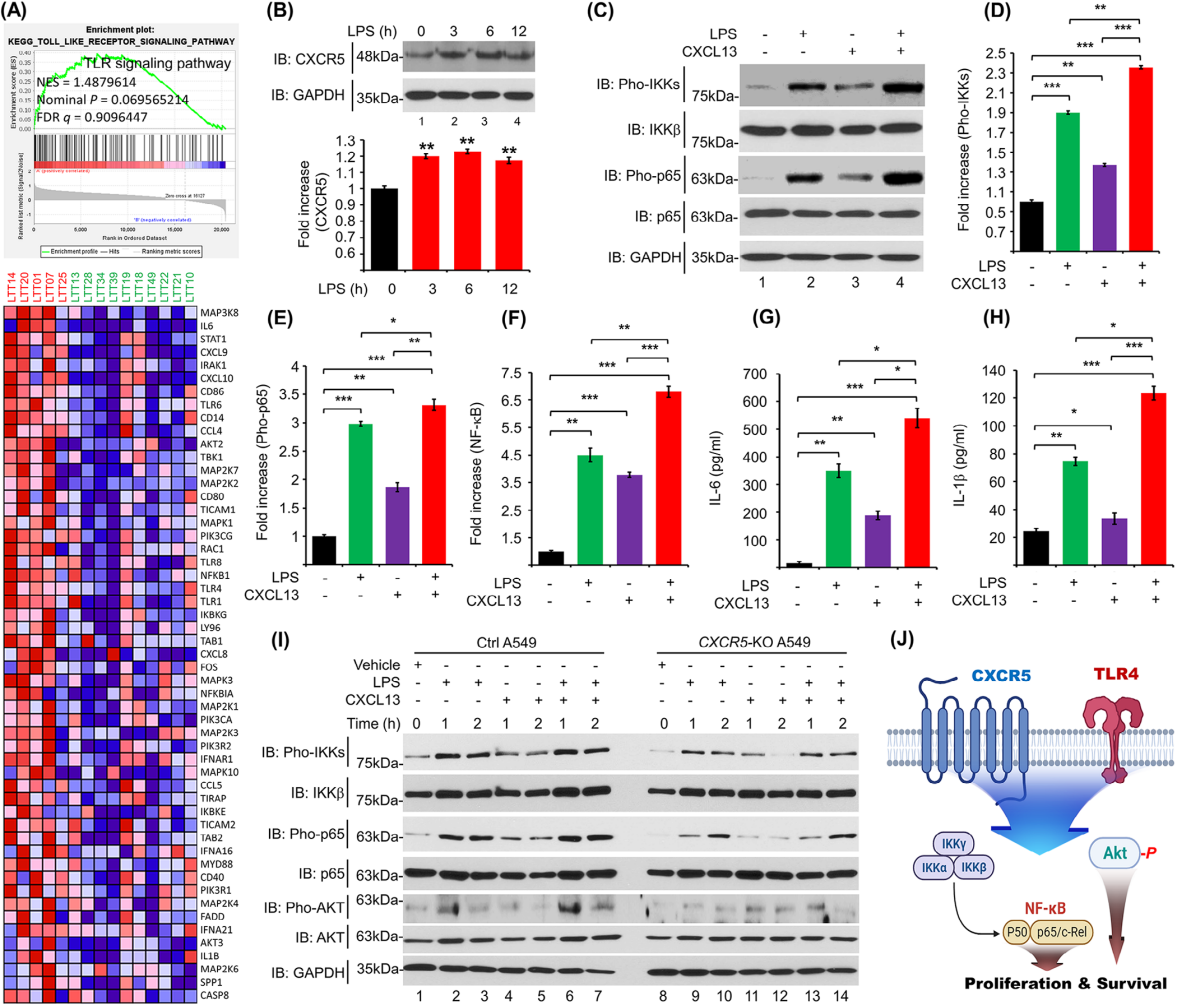
CXCR5 and TLR4 signal synergistically enhance the activation of NF‐κB in lung cancer cells. (**A**) GSEA was performed for five CXCR5^up^TLR4^up^ LTTs versus 10 CXCR5^down^TLR4^down^ LTTs. A gene set for the TLR signalling pathway is presented. A heat map of marker genes for the TLR signalling pathway is presented (down). NES, nominal *p* value and FDR *q* values are indicated in the inner panel. (B) A549 cells were treated with LPS (10 μg/mL) for different time periods as indicated. Endogenous CXCR5 levels were detected by western blotting with an anti‐CXCR5 antibody (upper). Levels of CXCR5 were quantified using ImageJ. Data are presented as mean ± SD of three independent experiments (down). (C–E) A549 cells were treated with LPS (10 μg/mL), CXCL13 (40 ng/mL) or LPS (10 μg/mL) plus CXCL13 (40 ng/mL) for 60 min. Phosphorylation levels of IKKs and p65 were measured by western blotting (C). Levels of pho‐IKKs (D) and pho‐p65 (E) were quantified using ImageJ. Data are presented as mean ± SD of three independent experiments. (F) A549 cells were transfected with a pBIIx‐luc NF‐κB‐dependent reporter construct and a Renilla luciferase vector and then treated with LPS (10 μg/mL), CXCL13 (40 ng/mL) or LPS (10 μg/mL) plus CXCL13 (40 ng/mL) for 24 h. Luciferase activity was measured. Results are presented as means ±  SD (*n* =  3) of three independent experiments. (G and H) A549 cells were treated with LPS (10 μg/mL), CXCL13 (40 ng/mL) or LPS (10 μg/mL) plus CXCL13 (40 ng/mL) for 24 h. Production levels of IL‐6 (G) and IL‐1β (H) in culture supernatant were measured by ELISA. Results are presented as mean ± SD of three independent experiments. (I) Ctrl A549 and *CXCR5*‐KO A549 cells were treated with vehicle (DMSO, 0.1% v/v concentration), LPS (10 μg/mL), CXCL13 (40 ng/mL) or LPS (10 μg/mL) plus CXCL13 (40 ng/mL) for different time periods as indicated. Western blotting analysis was performed with different antibodies as indicated. (J) A schematic model of how CXCR5 and TLR4 signal synergistically enhance the activation of NF‐κB and AKT. *, *p* < .05; **, *p* < .01; ***, *p* < .001.

**FIGURE 4:**
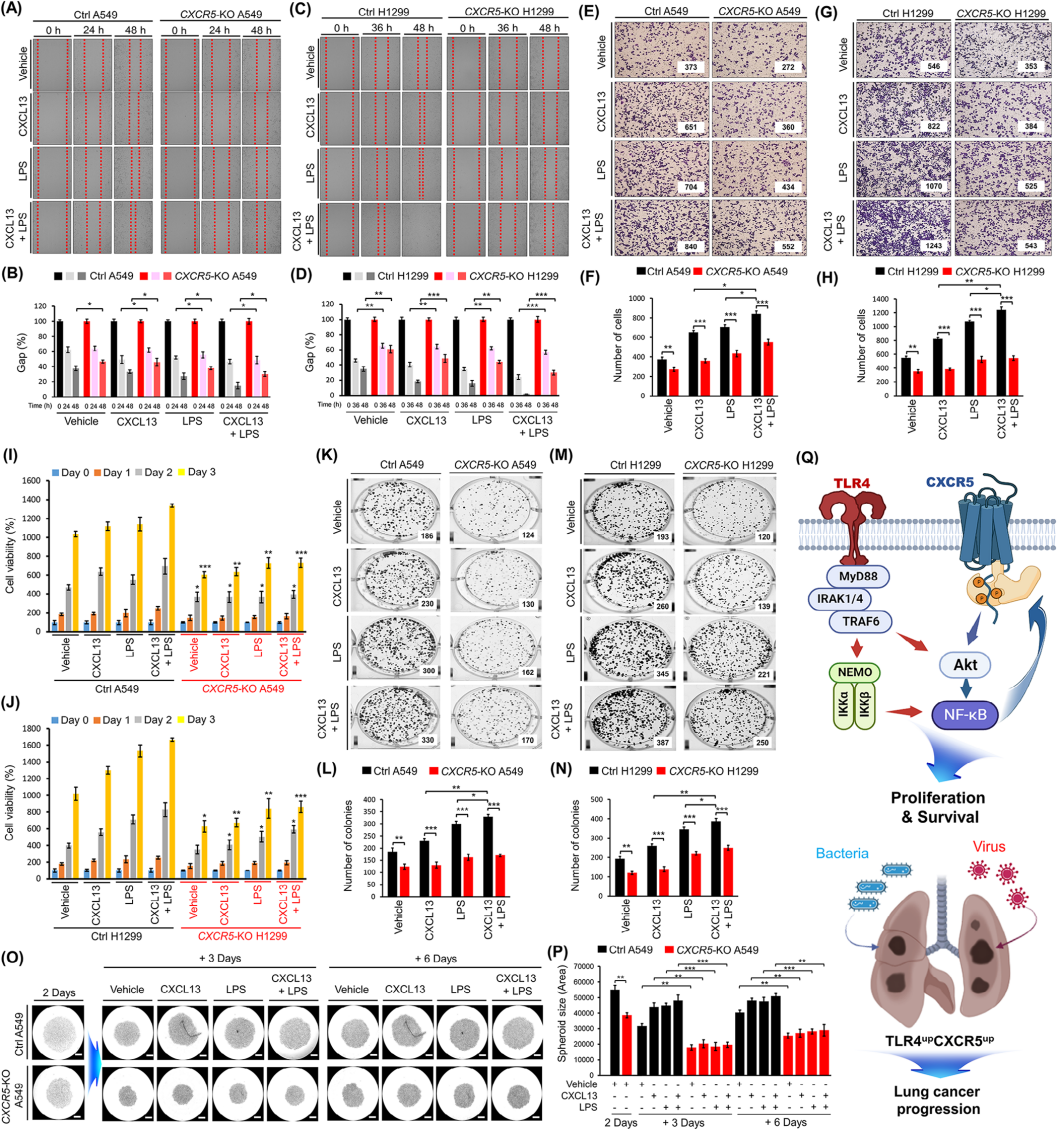
CXCR5 and TLR4 signals synergistically enhance lung cancer progression. (A–D) Ctrl A549 and *CXCR5*‐KO A549 cells (A and B) or Ctrl H1299 and *CXCR5*‐KO H1299 cells (C and D) were treated with vehicle (DMSO, 0.1% v/v concentration), CXCL13 (40 ng/mL), LPS (10 μg/mL) or CXCL13 (40 ng/mL) plus LPS (10 μg/mL) for different time periods as indicated. The residual gap between migrating cells from the opposing wound edge is expressed as a percentage of the initial scraped area (±SD, *n* = 3 different plates) (B and D). (E–H) Ctrl A549 and *CXCR5*‐KO A549 cells (E and F) or Ctrl H1299 and *CXCR5*‐KO H1299 cells (G and H) were treated with vehicle (DMSO, 0.1% v/v concentration), CXCL13 (30 ng/mL), LPS (15 μg/mL) or CXCL13 (30 ng/mL) plus LPS (15 μg/mL) for 24 h. The number of migrating cells was counted. Results are presented as mean ± SD of three independent experiments (F and H). (I and J) Ctrl A549 and *CXCR5*‐KO A549 cells (I) or Ctrl H1299 and *CXCR5*‐KO H1299 cells (J) were treated with vehicle (DMSO, 0.1% v/v concentration), CXCL13 (10 ng/mL), LPS (1 μg/mL) or CXCL13 (10 ng/mL) plus LPS (1 μg/mL) for different time periods as indicated. MTT assay was then performed as described in *Material and Methods*. Results are presented as mean ± SD of three independent experiments. *, *p* < .05; **, *p* < .01; and ***, *p* < .001 relative to respective Ctrl A549 or Ctrl H1299. (K–N) Ctrl A549 and *CXCR5*‐KO A549 cells (K and L) or Ctrl H1299 and *CXCR5*‐KO H1299 cells (M and N) were treated with vehicle (DMSO, 0.1% v/v concentration), CXCL13 (40 ng/mL), LPS (10 μg/mL) or CXCL13 (40 ng/mL) plus LPS (10 μg/mL) for 8 days. The number of colonies was counted. Results are presented as mean ± SD of three independent experiments (L and N). (O and P) The image of spheroids was represented in Ctrl A549 or *CXCR5*‐KO A549 cells treated with vehicle, CXCL13, LPS or CXCL13 plus LPS for different times (scale bar = 200 μm, 10× magnification, O). The spheroid growth as shown by the spheroid area was presented as mean ± SD (*n* = 5 spheroids) of three independent experiments (P). (Q) A schematic model of how CXCR5 and TLR4 signal synergistically enhance lung cancer progression. The expression of CXCR5 is up‐regulated by TLR4 signalling through activation of NF‐κB, consequently cooperating with CXCR5 and TLR4 signalling for NF‐κB and AKT activation. If accompanied by bacterial or viral infections, lung cancer patients with the up‐regulation of TLR4 and CXCR5 might be more likely to experience lung cancer progression. *, *p* < .05; **, *p* < .01; and ***, *p* < .001.

In summary, our results demonstrate that the expression of CXCR5 in lung tumour tissues of NSCLC patients is associated with cancer progression. Up‐regulated CXCR5, CXCL13 and TLR4 in lung tumour tissues were significantly enriched with gene sets regulating cancer formation and development, chemokine and innate signalling pathways. Importantly, *CXCR5*‐KO human lung cancer cells exhibited marked attenuations of cancer migration, proliferation and colony formation ability in response to CXCL13. In terms of functional aspects, the expression of CXCR5 was up‐regulated by TLR4 signalling through the activation of NF‐κB. Therefore, the lung cancer progressive ability was significantly elevated in response to CXCL13 and LPS, but markedly attenuated in *CXCR5*‐KO human lung cancer cells. As depicted in Figure 4Q, we propose a possible scenario in which the CXCR5–CXCL13 signalling axis is functionally implicated in lung cancer progression through a synergetic effect of TLR4 signalling. TLR4 signalling induces the production of CXCL13 and increases the expression of CXCR5 via activation of NF‐κB.^8^ Notably, it has been reported that bacterial infection is a potent cancer‐inducing factor that triggers cancer progression.^10^ Therefore, lung cancer patients with up‐regulation of TLR4 and CXCR5 might be expected to be more likely to experience NSCLC progression if they have bacterial or viral infections (Figure 4Q, down). Furthermore, it has been reported that TLR4 is strongly expressed in lung cancer tissues and associated with cancer progression, along with poor prognosis of patients with NSCLC.^7^ In addition, CXC chemokine ligand‐13 promotes metastasis via a CXCR5‐dependent signalling pathway in NSCLC,^2^ indicating a promising target for the prevention and inhibition of metastasis. Taken together, our clinically comparative results and functional investigations suggest that CXCL13/CXCR5 and TLR4 signals might be potential therapeutic targets capable of intervening NSCLCs in terms of clinical and application aspects.

## AUTHOR CONTRIBUTIONS

E. C. and K. Y. L. designed and supervised all experiments and contributed to the manuscript preparation. J. H. S., M. J. K., J. Y. K., Y. K. and S. K. J. performed the experiments and analysed the data. D. H. K., E. C. and K. Y. L. analysed TCGA and microarray data and contributed to the manuscript preparation. E. C. and K. Y. L. wrote the manuscript. All authors have read and approved the final manuscript.

## CONFLICT OF INTEREST STATEMENT

The authors declare that they have no competing interests.

## FUNDING INFORMATION

This work was supported by grants (2023R1A2C1003762, 2021R1A2C1094478 and RS‐2023‐00217189) of the National Research Foundation (NRF) funded by The Ministry of Science and ICT (MSIT), Republic of Korea.

## CONSENT FOR PUBLICATION

All authors agree to publish this article.

## ETHICS STATEMENT

All experiments were performed according to the Declaration of Helsinki and the study was approved by the Institutional Review Board (IRB) of Samsung Medical Center (SMC) (IRB#: 2010‐07‐204).

## Supporting information

Supporting information

## Data Availability

All data that support the findings of this study are available from the corresponding authors upon reasonable request.
