# Supplementary material for: CXCR5 and TLR4 signals synergistically enhance non‐small cell lung cancer progression
Source: Clin Transl Med. 2024 Jan 18;14(1):e1547. doi: 10.1002/ctm2.1547 (PMC10797246; doi:10.1002/ctm2.1547)
Supplement: Supplementary file 1 — Supporting information [file CTM2-14-e1547-s001.docx]

**Supporting Information**

CXCR5 and TLR4 signals synergistically enhance non-small cell lung cancer progression

**Methods and Materials**

**Non-small cell lung cancer (NSCLC) patients, tumors, and matched normal tissues**

This study was conducted in accordance with the ethical principles stated in the Declaration of Helsinki. It was approved by the Institutional Review Board (IRB#: 2010-07-204) of Samsung Medical Center (SMC, Seoul, Korea). Written informed consent to use pathological specimens for research was obtained from all patients prior to surgery. Lung tumor tissues and matched lung normal tissues of NSCLC patients (n = 42, Table S1), who have been diagnosed with lung cancer stage IA to stage IIIA, were obtained from SMC. Lung tumor and matched normal specimens of enrolled patients were immediately frozen in liquid nitrogen and stored at −80 °C until use. Lung tumor tissues and matched lung normal tissues were verified by the Department of laboratory medicine and Pathology at SMC.

**Cells**

A549 cells (human lung cancer cell line; CCL-185, American type culture collection (ATCC), Manassas, VA, USA) and H1299 cells (human non-small cell lung cancer cell line; CRL-5803, ATCC) were maintained in RPMI 1640 medium (LM011-01, Welgene, Daegu, Korea) supplemented with 10% fetal bovine serum (FBS), penicillin (100 μg/mL), and streptomycin (100 μg/mL) in a 5% CO_2_ humidified atmosphere at 37 °C.

**Antibodies and Reagents**

Anti-CXCR5 (sc-373775), anti-phospho-AKT1 (sc-52940), and anti-GAPDH (sc-47724) antibodies were purchased from Santa Cruz Biotechnology (Santa Cruz, CA, USA). Anti-phospho-NF-κB p65 (Ser536, 93H1, 3033), anti-NF-κB p65 (D14E12, 8242), anti-phospho-IKKs (Ser176/180, 16A6, 2697S), anti-AKT1 (D9R8K, 75692), and anti-IKKβ (2684S) antibodies were purchased from Cell Signaling Technology (Danvers, MA, USA). Goat anti-rabbit IgG (HRP) (GTX213110-01) antibody was purchased from GeneTex Inc. (Irvine, CA, USA). Rabbit anti-mouse IgG H&L (HRP) (ab6728) antibody was purchased from Abcam (Cambridge, MA, USA). Recombinant Human CXCL13/BLC/BCA-1 Protein (CXCL13; NBP2-34885) was purchased from Novus Biologicals (Littleton, CO, USA). Lipopolysaccharide (LPS; L3024), Dimethyl sulfoxide (DMSO; D4540), Dulbecco’s phosphate-buffered saline (DPBS; D8537), Glutaraldehyde (G6257-100ml), Crystal violet (C6158-50g), and Thiazolyl Blue Tetrazolium Bromide (MTT; M5655) were purchased from Sigma-Aldrich (St Louis, MO, USA). Lipofectamine 2000 (11668019) was purchased from Thermo Fisher Scientific (Waltham, MA, USA).

**Generation of *CXCR5*-knockout (*CXCR5*-KO) Cell Line with CRISPR/Cas9**

To generate *CXCR5-*KO lung cancer cells with CRISPR/Cas9 gene editing method, we used two vector systems including single guide RNA (sgRNA) and CRISPR-associated protein 9 (Cas9) vectors, as previously described.^1^ sgRNA and Cas9 vectors were kindly provided by Dr. Daesik Kim (Sungkyunkwan University School of Medicine, Suwon, Korea). Guide RNA sequences for CRISPR/Cas9 were designed on the CRISPR design website (http://crispr.mit.edu/), provided by the Feng Zhang Lab. Insert oligonucleotides for human CXCR5 gRNA were 5’-GATCGGCAACGTCCTGGTGC-3’ (gRNA-1) / 5’-CATGAACTACCCGCTAACGC-3’ (gRNA-2) / 5’-GCGTGAAGTTCCGCAGTGAC-3’ (gRNA-3). Complementary oligonucleotides to guide RNAs (gRNAs) were annealed and cloned into a sgRNA vector. sgRNA vector expressing gRNA of CXCR5 and Cas9 vector expressing Cas9 were transfected into A549 cells and H1299 cells using Lipofectamine 2000 (Thermo Fisher Scientific, Waltham, MA, USA) according to the manufacturer’s instructions. After two weeks, colonies were isolated from 96-well plates, and expression levels of CXCR5 were analyzed with western blotting.

**Western Blotting (WB) Assays**

Control (Ctrl) A549 lung cancer cells were seeded into 12-well plates and cultured. Cells were treated with vehicle (DMSO, 0.1 % v/v concentration), LPS (10 μg/mL), CXCL13 (40 ng/mL), or co-treatment of LPS (10 μg/mL) and CXCL13 (40 ng/mL) for different times. After collecting the cells, cell lysates were separated by sodium dodecyl sulfate-polyacrylamide gel electrophoresis (SDS-PAGE, 8~12 %) and immune-probed with anti-CXCR5, anti-phospho-p65, anti-p65, anti-phospho-IKKs, anti-IKKβ, or anti-GAPDH antibodies (as the loading control). Ctrl A549, *CXCR5*-KO A549, Ctrl H1299, and *CXCR5*-KO H1299 lung cancer cells were seeded into 12-well plates and cultured. Cells were treated with vehicle (DMSO, 0.1 % v/v concentration), LPS (10 μg/mL), CXCL13 (40 ng/mL), or co-treatment of LPS (10 μg/mL) and CXCL13 (40 ng/mL) for different times. Cell lysates were separated by SDS-PAGE (8~12 %) and immune-probed with anti-phospho-p65, anti-p65, anti-phospho-IKKs, anti-IKKβ, anti-AKT, anti-phospho-AKT, or anti-GAPDH antibodies (as the loading control).

**Wound-Healing Migration Assay**

A wound-healing migration assay was performed following previous protocols.^2-5^ Briefly, Control (Ctrl) A549, *CXCR5*-KO A549, Ctrl H1299, and *CXCR5*-KO H1299 cells were seeded into 12-well plates and cultured to reach confluence. Cell monolayers were gently scratched and washed with a culture medium. After floating cells and debris were removed, cells attached to the culture plates were treated with vehicle (DMSO, 0.1% v/v concentration), LPS (10 μg/mL), CXCL13 (40 ng/mL), or co-treatment of LPS (10 μg/mL) and CXCL13 (40 ng/mL) for different times. Cell images were captured after culturing for different time periods as indicated in each experiment.

**Transwell Migration Assay**

Control (Ctrl) A549, *CXCR5*-KO A549, Ctrl H1299, and *CXCR5*-KO H1299 lung cancer cells were suspended in a culture medium (250 μL) and added to the upper compartment of a 24-well Transwell® chamber (8 μm pore; Corning, 3422). Ctrl A549, *CXCR5*-KO A549 lung cancer cells and culture medium (250 μL) were mixed with vehicle (DMSO, 0.1 % v/v concentration), LPS (15 μg/mL), CXCL13 (30 ng/mL), or co-treatment of LPS (15 μg/mL) and CXCL13 (30 ng/mL) and incubated at 37 °C for 24 h. Ctrl H1299, and *CXCR5*-KO H1299 lung cancer cells and culture medium (250 μL) were mixed with vehicle (DMSO, 0.1 % v/v concentration), LPS (10 μg/mL), CXCL13 (20 ng/mL), or co-treatment of LPS (10 μg/mL) and CXCL13 (20 ng/mL) and incubated at 37 °C for 24 h. Migratory cells pass through polycarbonate membrane and cling to the bottom side. Non-migratory cells stay in the upper chamber. After removal of non-migratory cells, migratory cells were fixed using 2.5 % glutaraldehyde (Sigma-Aldrich, G6257-100 mL) and then stained with 0.1% crystal violet (Sigma-Aldrich, C6158-50g).

**Anchorage-independent Soft Agar Colony Formation Assay**

Anchorage-independent soft agar colony formation assay was performed following previous protocols.^3,6^ Briefly, Control (Ctrl) A549, *CXCR5*-KO A549, Ctrl H1299, and *CXCR5*-KO H1299 cells (1 × 10^4^ cells /well) mixed with 0.3% Agarose (Biotechnolony Grade, GA001.500) in complete medium were plated on bottom of a 0.5% agar layer in a 6-well plate with a complete medium. Growth medium (2 mL) with vehicle (DMSO, 0.1% v/v concentration), LPS (10 μg/mL), CXCL13 (20 ng/mL), or co-treatment of LPS (10 μg/mL) and CXCL13 (20 ng/mL) was added on top of the layer and the cells were incubated at 37 °C for 24 days or 35 days.

**Colony Formation Assay**

The ability of a single cell to grow into a colony was passed through a colony formation assay as previously described.^3, 7, 8^ Control (Ctrl) A549, *CXCR5*-KO A549, Ctrl H1299, and *CXCR5*-KO H1299 cells were harvested with trypsin-EDTA and resuspended in a singular form. The 1 × 10^3^ cells/well were plated in a 6-well plate and treated with a vehicle (DMSO, 0.1% v/v concentration), LPS (10 μg/mL), CXCL13 (40 ng/mL), or co-treatment of LPS (10 μg/mL) and CXCL13 (40 ng/mL). After incubation for ~8 days, colonies were stained with 0.5% crystal violet (Sigma-Aldrich, C6158-50g) for 30 min at room temperature.

**3D Spheroids formation assay using agarose-coated plates**

3D Spheroids formation assay was performed following previous protocols.^9^ 1.5% agarose hydrogel was added to each well of a 96-well culture plate, and incubated at room temperature (RT) for 30 min. Ctrl A549 or *CXCR5*-KO A549 cells were seeded in 100 µl growth medium at a concentration of 10,000 cells per well. Plates were incubated at 37 °C for an additional 48 hours to allow formation of 3D spheroids in culture. The spheroid was added with vehicle, CXCL13 (40 ng/mL), LPS (10 μg/mL), or CXCL13 (40 ng/mL) plus LPS (10 μg/mL), and incubated for additional times. Spheroid formation and growth can be evaluated using phase-contrast microscopy. The size of the spheroids was assessed using the Image J Software (National Institutes of Health, Bethesda, MD, USA).

**MTT Assay**

Control (Ctrl) A549, *CXCR5*-KO A549, Ctrl H1299, and *CXCR5*-KO H1299 cells were seeded in a 96-well culture plate at a density of 1 × 10^3^ cells/well, treated with vehicle (DMSO, 0.1% v/v concentration), LPS (1 μg/mL), CXCL13 (10 ng/mL), or co-treatment of LPS (1 μg/mL) and CXCL13 (10 ng/mL), and grown in a culture medium supplemented with 10% FBS for different times. Cell viability was measured using an MTT reagent (Sigma-Aldrich, M5655) dissolved in PBS (1 mg/mL). On the day when the measurements were taken, the medium was carefully replaced on fresh RPMI + 10% FBS with diluted MTT (1:10, 10% MTT) and incubated for 3 h at 37 °C. After removing the incubation medium, formazan crystals were dissolved in a 100 μl solution of DMSO. MTT reduction was quantified by measuring the light absorbance at 595 nm using the Bio-Rad Model 680 microplate reader (Bio-Rad, CA, USA). Each test was repeated at least four times in the quadruple.

**NF-κB luciferase reporter assay**

Luciferase reporter assay was performed as previously described.^10^ Briefly, Control (Ctrl) A549, *CXCR5*-KO A549, Ctrl H1299, and *CXCR5*-KO H1299 cells were transfected with mock, pBIIx-luc NF-κB-dependent reporter construct, Renilla luciferase vector (Promega, Madison, WI, USA). At 24 h post-transfection, cells were treated with vehicle (DMSO, 0.1% v/v concentration), LPS (10 μg/mL), CXCL13 (40 ng/mL), or co-treatment of LPS (10 μg/mL) and CXCL13 (40 ng/mL) for 24 h and lysed. Luciferase activity was measured using a dual luciferase assay kit (Promega, 72050).

**Enzyme-linked immunosorbent assay (ELISA)**

A549 cells were plated in 24-well culture plates at 2 × 10^5^ cells/well and treated with vehicle (DMSO, 0.1% v/v concentration), LPS (10 μg/mL), CXCL13 (40 ng/mL), or co-treatment of LPS (10 μg/mL) and CXCL13 (40 ng/mL) for 24 h. The interleukin (IL)-6 and IL-1β levels in the culture supernatant were determined by ELISA using the ELISA development kit (R&D Systems, Inc., Minneapolis, MN, USA) according to the manufacturer's instructions.

**Microarray Analysis**

Microarray analysis was performed as previously described.^11-14^ From tumor and matched normal tissues of 42 patients with NSCLC, total RNAs were extracted with Trizol (Thermo Fisher Scientific, 15596026) and purified using RNeasy columns (Qiagen, 74106) according to each manufacturer’s protocol.

**Gene Set Enrichment Analysis (GSEA)**

Different magnitudes (∆ Mags) of CXCL13, CXCR5, and TLR4 expression were obtained from pre-processed microarray data between lung tumor tissues and matched lung normal tissues. Fourteen CXCR5^up^CXCL13^up^ LTTs and seven CXCR5^down^CXCL13^down^ LTTs or five CXCR5^up^TLR4^up^ LTTs and ten CXCR5^down^TLR4^down^ LTTs were selected based on different magnitudes of CXCL13, CXCR5, and TLR4. Genes showing significant differences, such as normalized enrichment score (NES), nominal *P*-value, and false discovery rate (FDR) *q*-values, were analyzed by GSEA (<http://www.gsea-msigdb.org/gsea/index.jsp>).

**TCGA Data Analysis**

The expression of CXCL13 in human lung cancer was analyzed using TCGA data (GEPIA, gene expression profiling interactive analysis; http://gepia.cancer-pku.cn/detail.php?gene=CXCL13###).

**Statistical Analysis**

All data are expressed as mean ± SD (standard deviation). Statistical significance was determined by Student’s t-test using GraphPad Prism 5.0 (GraphPad Software, San Diego, CA, USA). *P*-values were marked as **P* < 0.05, ***P* < 0.01, and ****P* < 0.001.

**References**

1. Cong L, Ran FA, Cox D, Lin S, Barretto R, Habib N, et al. Multiplex genome engineering using CRISPR/Cas systems. *Science*. 2013;**339**(6121):819-23.

2. Min Y, Kim MJ, Lee S, Chun E, Lee KY. Inhibition of TRAF6 ubiquitin-ligase activity by PRDX1 leads to inhibition of NFKB activation and autophagy activation. *Autophagy.* 2018; **14**(8):1347-1358.

3. Son J, Kim MJ, Lee JS, Kim JY, Chun E, Lee KY. Hepatitis B virus X Protein Promotes Liver Cancer Progression through Autophagy Induction in Response to TLR4 Stimulation. *Immune Netw.* 2021; **21**(5):e37.

4. Kim MJ, Min Y, Im JS, Son J, Lee JS, Lee KY. p62 is Negatively Implicated in the TRAF6-BECN1 Signaling Axis for Autophagy Activation and Cancer Progression by Toll-Like Receptor 4 (TLR4). *Cells.* 2020; **9**(5):1142.

5. Kim MJ, Min Y, Shim JH, Chun E, Lee KY. CRBN Is a Negative Regulator of Bactericidal Activity and Autophagy Activation Through Inhibiting the Ubiquitination of ECSIT and BECN1. *Front Immunol.* 2019; **10**:2203.

6. Borowicz S, Van Scoyk M, Avasarala S, et al. The soft agar colony formation assay. *J Vis Exp.* 2014; (92):e51998.

7. Park S, Ha YN, Dezhbord M, et al. Suppression of Hepatocyte Nuclear Factor 4 α by Long-term Infection of Hepatitis B Virus Contributes to Tumor Cell Proliferation. *Int J Mol Sci.* 2020; **21**(3):948.

8. Franken NA, Rodermond HM, Stap J, Haveman J, van Bree C. Clonogenic assay of cells in vitro. *Nat Protoc.* 2006; **1**(5):2315-2319.

9. Shoval H, Karsch-Bluman A, Brill-Karniely Y, Stern T, Zamir G, Hubert A, Benny O. Tumor cells and their crosstalk with endothelial cells in 3D spheroids. *Sci Rep.* 2017; **7**(1):10428.

10. Wi SM, Moon G, Kim J, et al. TAK1-ECSIT-TRAF6 complex plays a key role in the TLR4 signal to activate NF-κB. *J Biol Chem.* 2014; **289**(51):35205-35214.

11. Kim MJ, Min Y, Son J, et al. AMPKα1 Regulates Lung and Breast Cancer Progression by Regulating TLR4-Mediated TRAF6-BECN1 Signaling Axis. *Cancers (Basel).* 2020; **12**(11):3289.

12. Min Y, Wi SM, Shin D, Chun E, Lee KY. Peroxiredoxin-6 Negatively Regulates Bactericidal Activity and NF-κB Activity by Interrupting TRAF6-ECSIT Complex. *Front Cell Infect Microbiol.* 2017; **7**:94.

13. Kim SY, Jeong S, Jung E, et al. AMP-activated protein kinase-α1 as an activating kinase of TGF-β-activated kinase 1 has a key role in inflammatory signals. *Cell Death Dis.* 2012; **3**(7):e357.

14. Min Y, Wi SM, Kang JA, et al. Cereblon negatively regulates TLR4 signaling through the attenuation of ubiquitination of TRAF6. *Cell Death Dis.* 2016; **7**(7):e2313.

**Supplementary figures and figure legends**

**Figure S1**

**
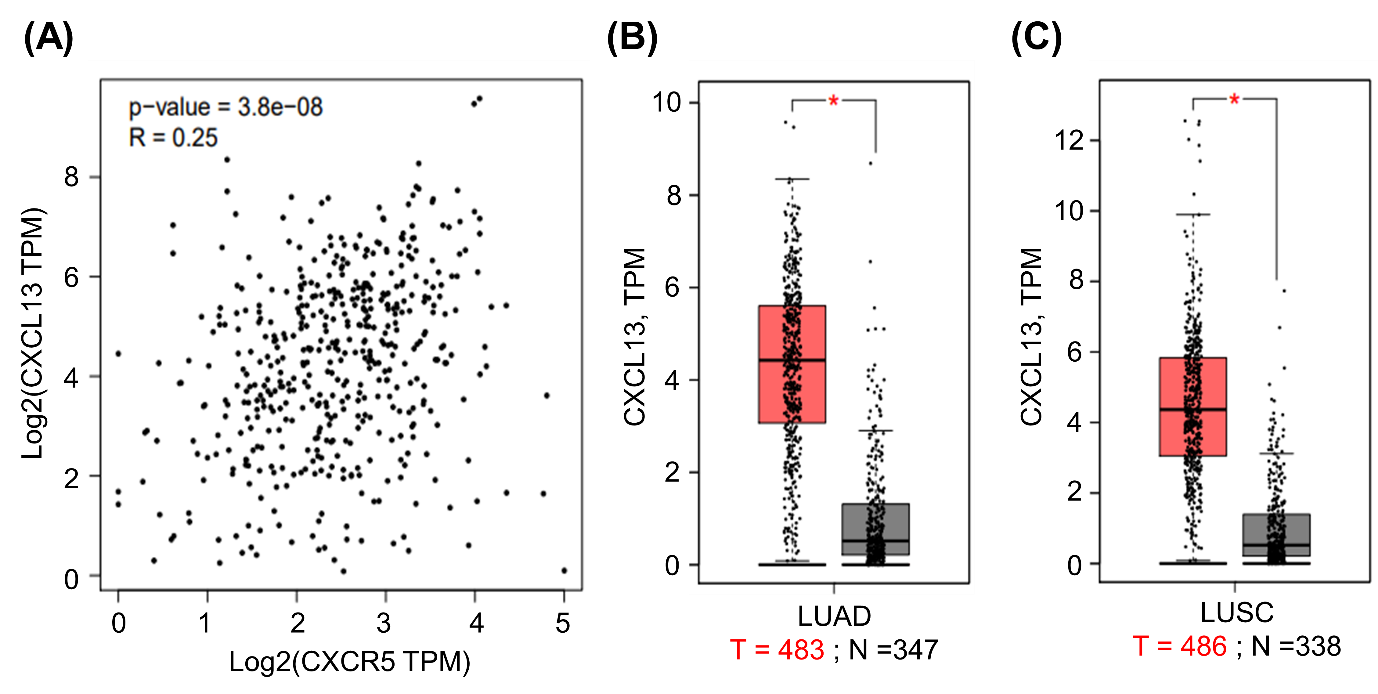
**

**Figure S1. Expression of CXCL13 and CXCR5 in lung cancer tissues and normal tissues from GEPIA.** (**A**) The gene expression correlation between CXCL13 and CXCR5 in lung adenocarcinoma (LUAD) from GEPIA (http://gepia.cancer-pku.cn/detail.php?gene=CXCL13###). (**B** and **C**) Expression of CXCL13 in LUAD (**B**) and lung squamous cell carcinoma (LUSC) (**C**).

**Figure S2**

**
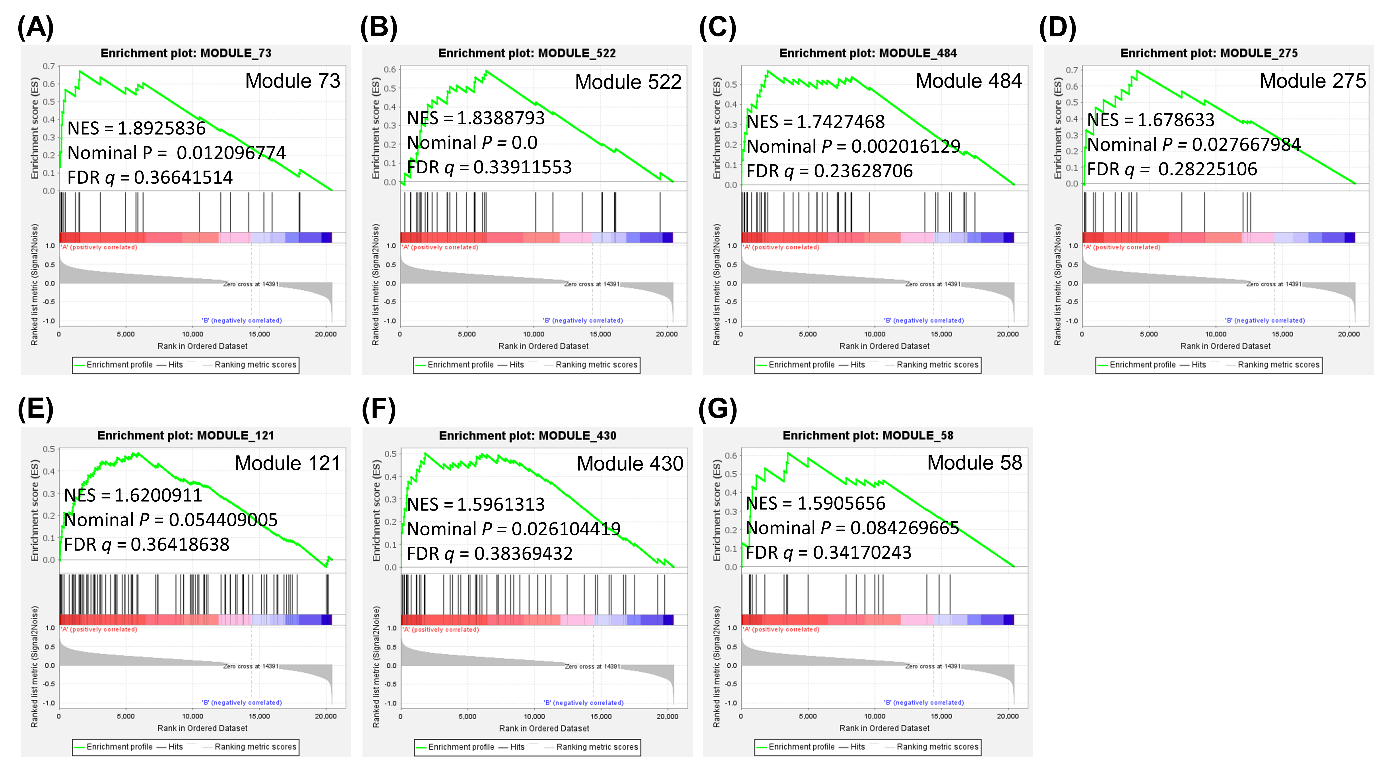
**

**Figure S2. Gene set enrichment analysis (GSEA) between fourteen CXCR5^up^CXCL13^up^ LTTs and seven CXCR5^down^CXCL13^down^ LTTs. (A-G).** 42 lung tumor tissues of NSCLC patients were listed according to the different magnitudes (∆Mag) of ∆CXCR5 and ∆CXCL13 expression in lung tumor tissues (LTTs) vs. matched lung normal tissues (mLNTs). Fourteen CXCR5^up^CXCL13^up^ LTTs (red boxes) and seven CXCR5^down^CXCL13^down^ LTTs (green boxes) were selected for GSEA (Figure 1A). GSEA (<https://www.gsea-msigdb.org/gsea/index.jsp>) was performed in fourteen CXCR5^up^CXCL13^up^ LTTs vs. seven CXCR5^down^CXCL13^down^ LTTs. Gene sets for seven cancer modules are presented. NES, nominal *P*-value, and FDR *q*-values are indicated in the inner panel.

**Figure S3**


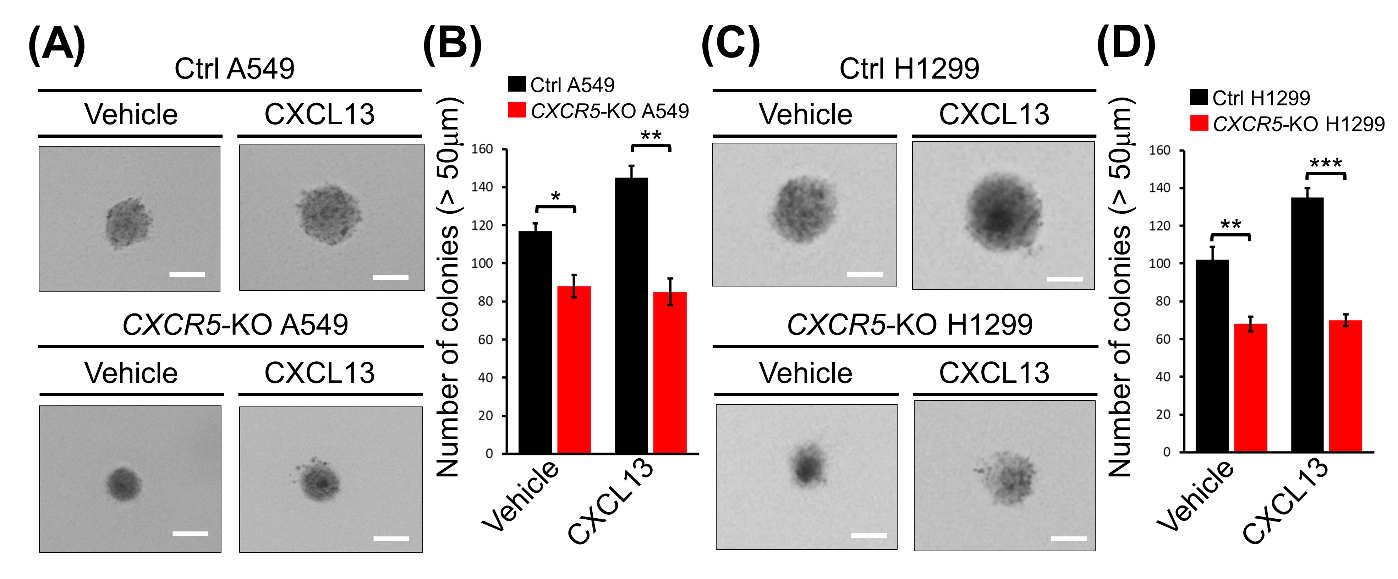


**Figure S3. Anchorage-independent colony formation assay in Ctrl A549 and *CXCR5*-KO A549 or Ctrl H1299 and *CXCR5*-KO H1299 in response to CXCL13.** Ctrl A549 and *CXCR5*-KO A549 cells (**A** and **B**) or Ctrl H1299 and *CXCR5*-KO H1299 cells (**C** and **D**) were treated with vehicle (DMSO, 0.1% v/v concentration) and CXCL13 (20 ng/mL) for 24 days or 35 days, respectively. The number of colonies was counted. Results are presented as the mean ± SD of three independent experiments. Scale bar, 50 μm. * *P* < 0.05, ** *P* < 0.01, and *** *P* < 0.001.

**Figure S4**

**
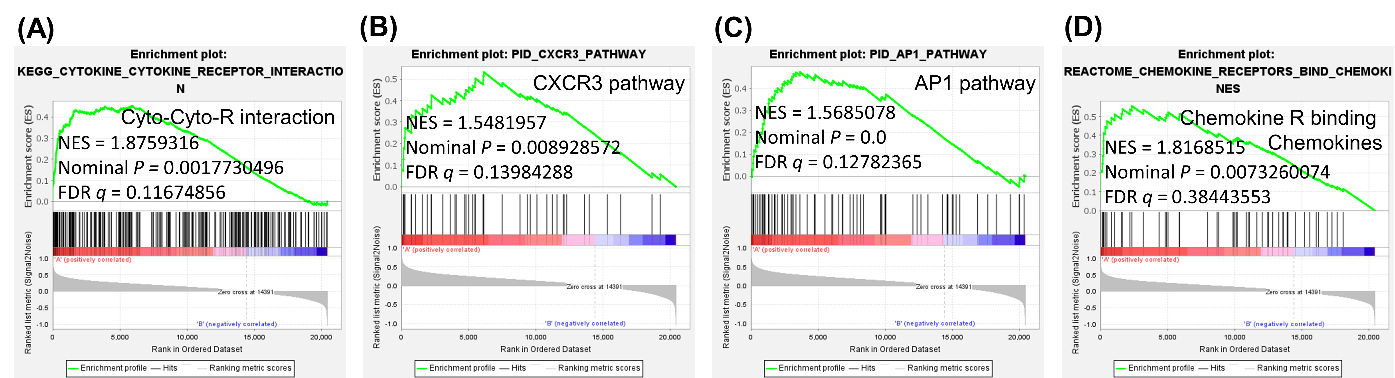
**

**Figure S4. Gene set enrichment analysis (GSEA) between fourteen CXCR5^up^CXCL13^up^ LTTs and seven CXCR5^down^CXCL13^down^ LTTs. (A-D).** 42 lung tumor tissues of NSCLC patients were listed according to the different magnitudes (∆Mag) of ∆CXCR5 and ∆CXCL13 expression in lung tumor tissues (LTTs) vs. matched lung normal tissues (mLNTs). Fourteen CXCR5^up^CXCL13^up^ LTTs (red boxes) and seven CXCR5^down^CXCL13^down^ LTTs (green boxes) were selected for GSEA (Figure 1A). GSEA (<https://www.gsea-msigdb.org/gsea/index.jsp>) was performed in fourteen CXCR5^up^CXCL13^up^ LTTs vs. seven CXCR5^down^CXCL13^down^ LTTs. Gene sets for cytokine-cytokine receptor interaction (**A**), CXCR3 pathway (**B**), AP1 pathway (**C**), and chemokine receptor binding chemokines (**D**) are presented. NES, nominal *P*-value, and FDR *q*-values are indicated in the inner panel.

**Figure S5**

**
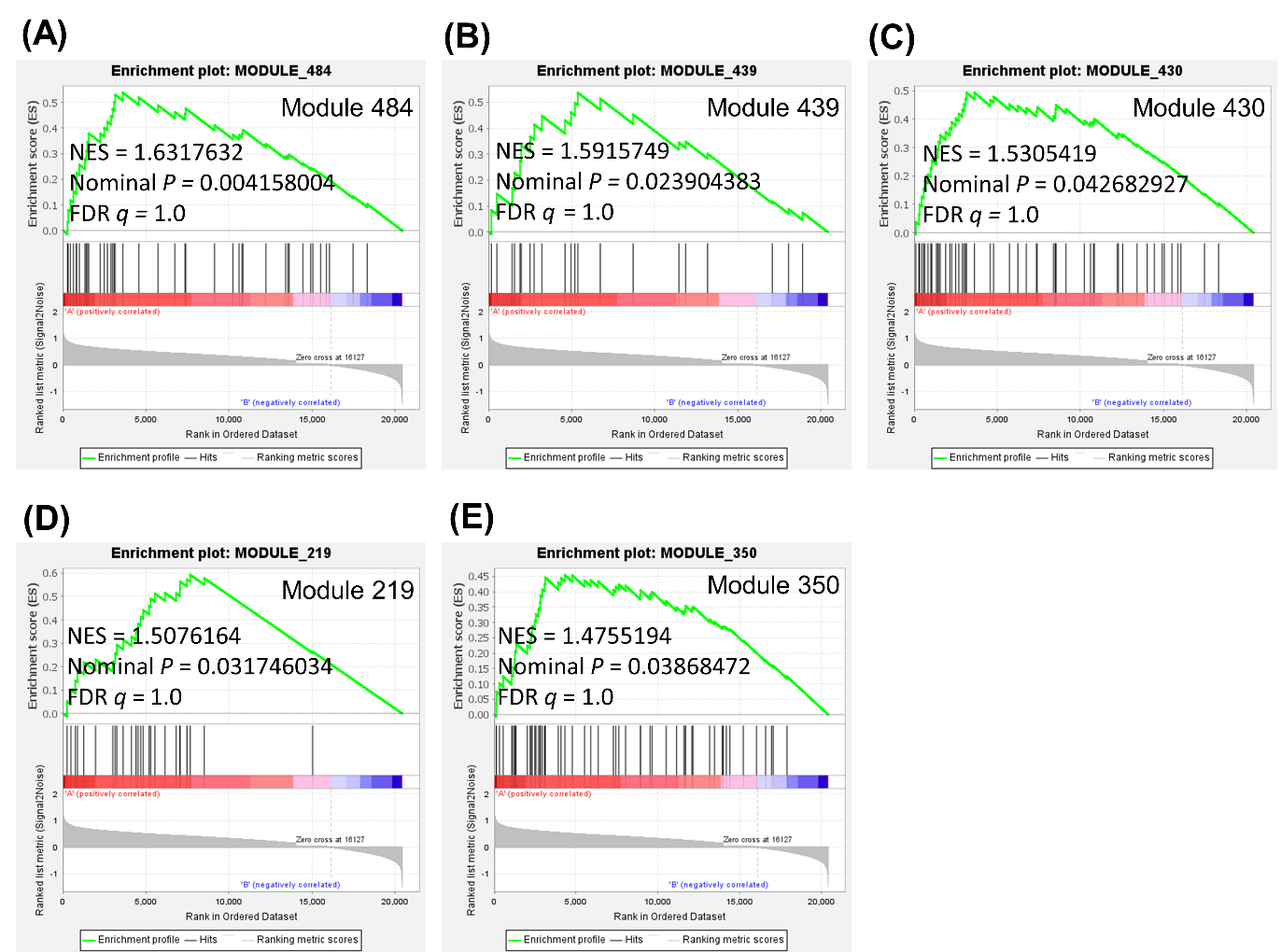
**

**Figure S5. Gene set enrichment analysis (GSEA) between five CXCR5^up^TLR4^up^ LTTs and ten CXCR5^down^TLR4^down^ LTTs. (A-E).** 42 lung tumor tissues of NSCLC patients were listed according to the different magnitudes (∆Mag) of ∆TLR4 and ∆CXCR5 expression in lung tumor tissues (LTTs) vs. matched lung normal tissues (mLNTs). Five CXCR5^up^TLR4^up^ LTTs and ten CXCR5^down^TLR4^down^ LTTs were selected for GSEA. GSEA (<https://www.gsea-msigdb.org/gsea/index.jsp>) was performed in five CXCR5^up^TLR4^up^ LTTs vs. ten CXCR5^down^TLR4^down^ LTTs. Gene sets for five cancer modules are presented. NES, nominal *P*-value, and FDR *q*-values are indicated in the inner panel.

**Figure S6**


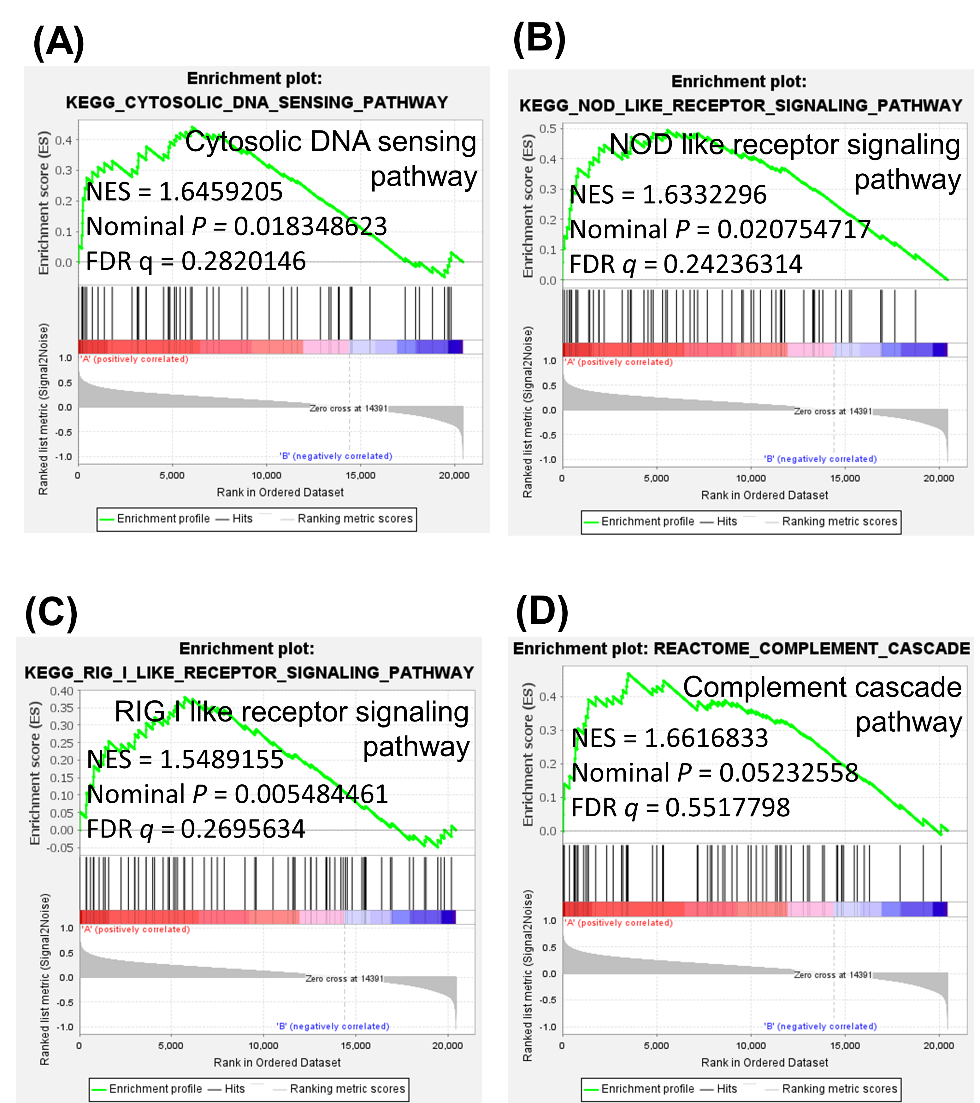


**Figure S6. Gene set enrichment analysis (GSEA) between five CXCR5^up^TLR4^up^ LTTs and ten CXCR5^down^TLR4^down^ LTTs. (A-D).** 42 lung tumor tissues of NSCLC patients were listed according to the different magnitudes (∆Mag) of ∆TLR4 and ∆CXCR5 expression in lung tumor tissues (LTTs) vs. matched lung normal tissues (mLNTs). Five CXCR5^up^TLR4^up^ LTTs and ten CXCR5^down^TLR4^down^ LTTs were selected for GSEA. GSEA (<https://www.gsea-msigdb.org/gsea/index.jsp>) was performed in five CXCR5^up^TLR4^up^ LTTs vs. ten CXCR5^down^TLR4^down^ LTTs. Gene sets for cytosolic DNA sensing pathway (**A**), NOD-like receptor signaling pathway (**B**), RIG-1 like receptor signaling pathway (**C**), and complement cascade pathway (**D**) are presented. NES, nominal *P*-value, and FDR *q*-values are indicated in the inner panel.

**Figure S7**


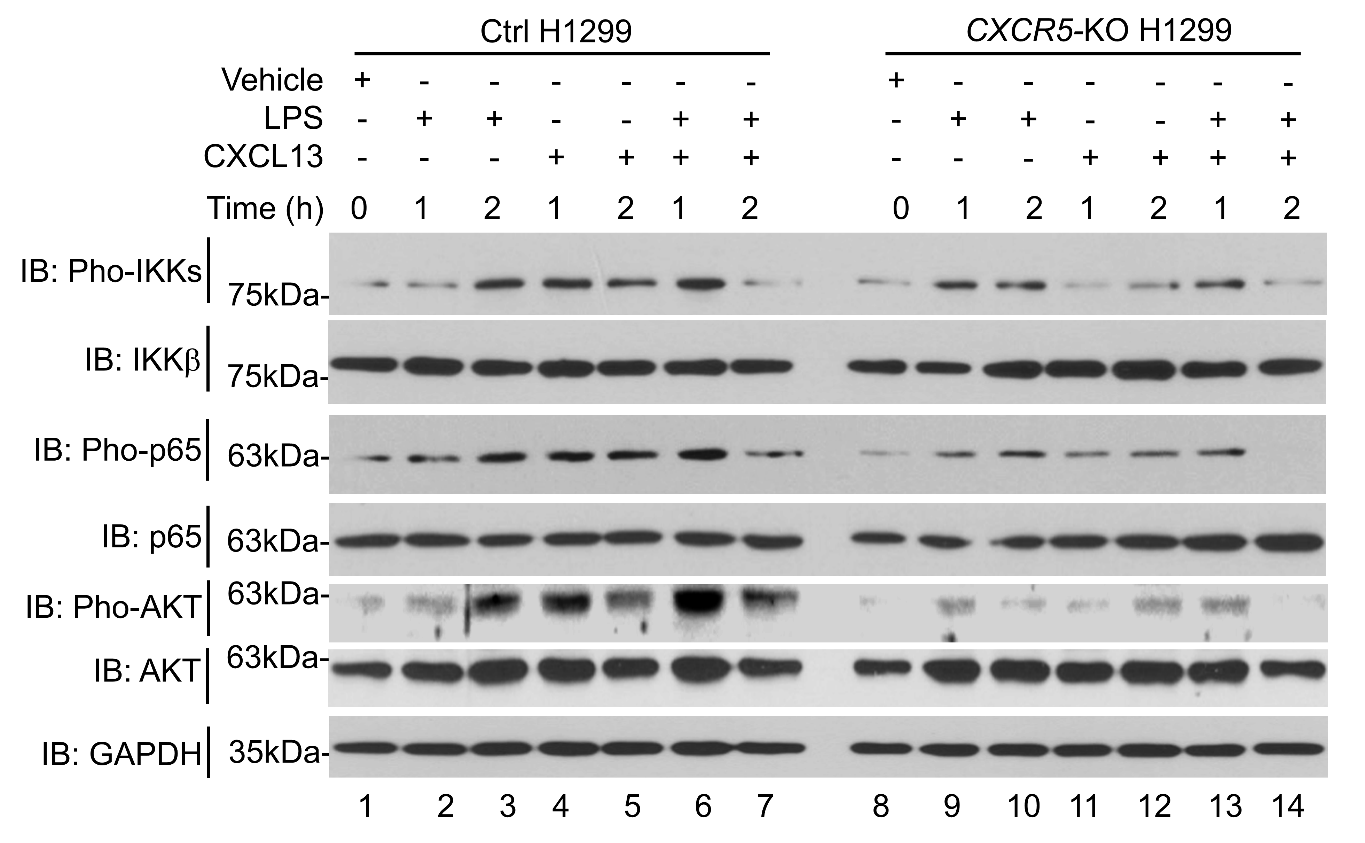


**Figure S7. Western blotting analysis in Ctrl H1299 and *CXCR5*-KO H1299 treated with vehicle, LPS, CXCL13, or co-treatment of LPS and CXCL13.** Ctrl H1299 and *CXCR5*-KO H1299 cells were treated with vehicle (DMSO, 0.1% v/v concentration), LPS (10 μg/mL), CXCL13 (40 ng/mL), or co-treatment of LPS (10 μg/mL) and CXCL13 (40 ng/mL) for different times, as indicated. Cell lysates were prepared and immune-probed with anti-pho-IKKs, anti-IKKβ, anti-pho-p65, anti-p65, anti-pho-AKT, anti-AKT, and anti-GAPDH antibodies, as indicated.

**Figure S8**

**
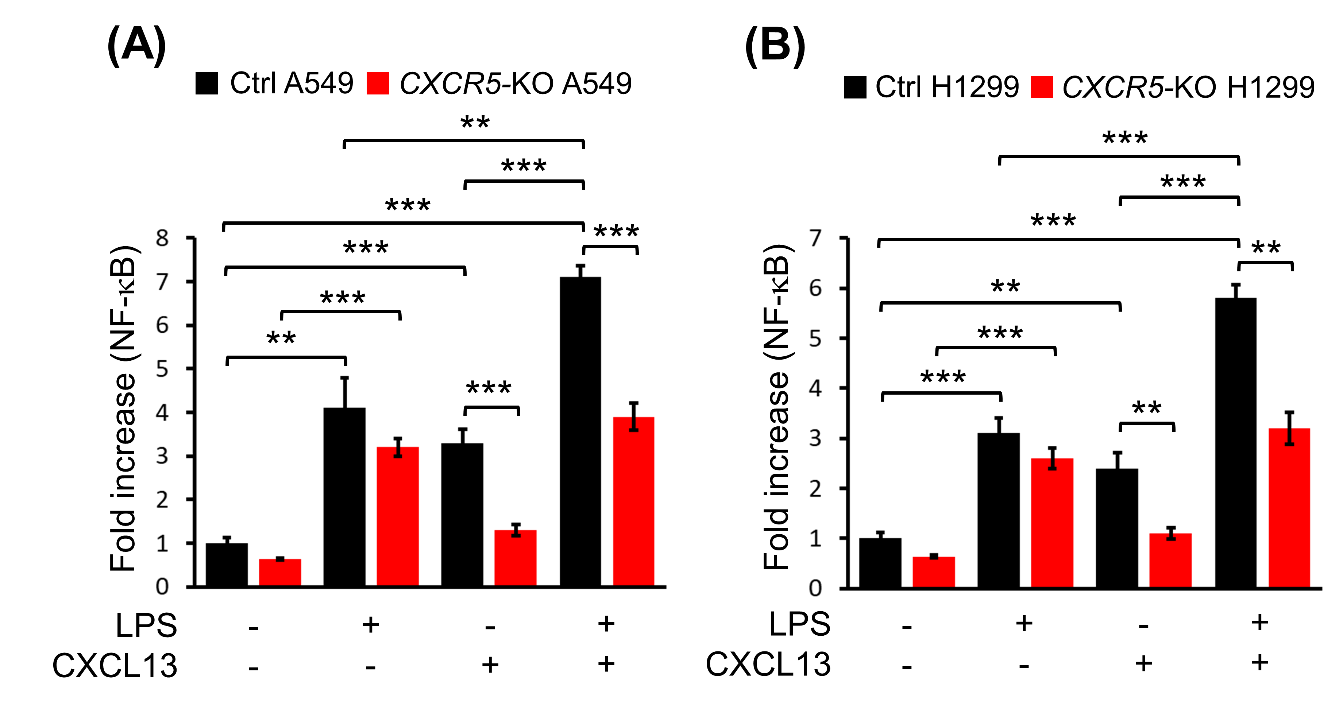
**

**Figure S8. Luciferase assay for NF-κB in Ctrl A549 and *CXCR5*-KO A549 or Ctrl H1299 and *CXCR5*-KO H1299 treated with vehicle, LPS, CXCL13, or co-treatment of LPS and CXCL13.** (**A** and **B**) Control (Ctrl) A549 and *CXCR5*-KO A549 cells (**A**) or Ctrl H1299 and *CXCR5*-KO H1299 cells (**B**) were transfected with the pBIIx-luc NF-κB-dependent reporter construct and the Renilla luciferase vector, and treated with vehicle (DMSO, 0.1% v/v concentration), LPS (10 μg/mL), CXCL13 (40 ng/mL), or co-treatment of LPS (10 μg/mL) and CXCL13 (40 ng/mL) for 24 h. Luciferase activity was measured. Results are means ± SD (n = 3) of three independent experiments. ***P*<0.01 and ****P* <0.001.

**Figure S9**


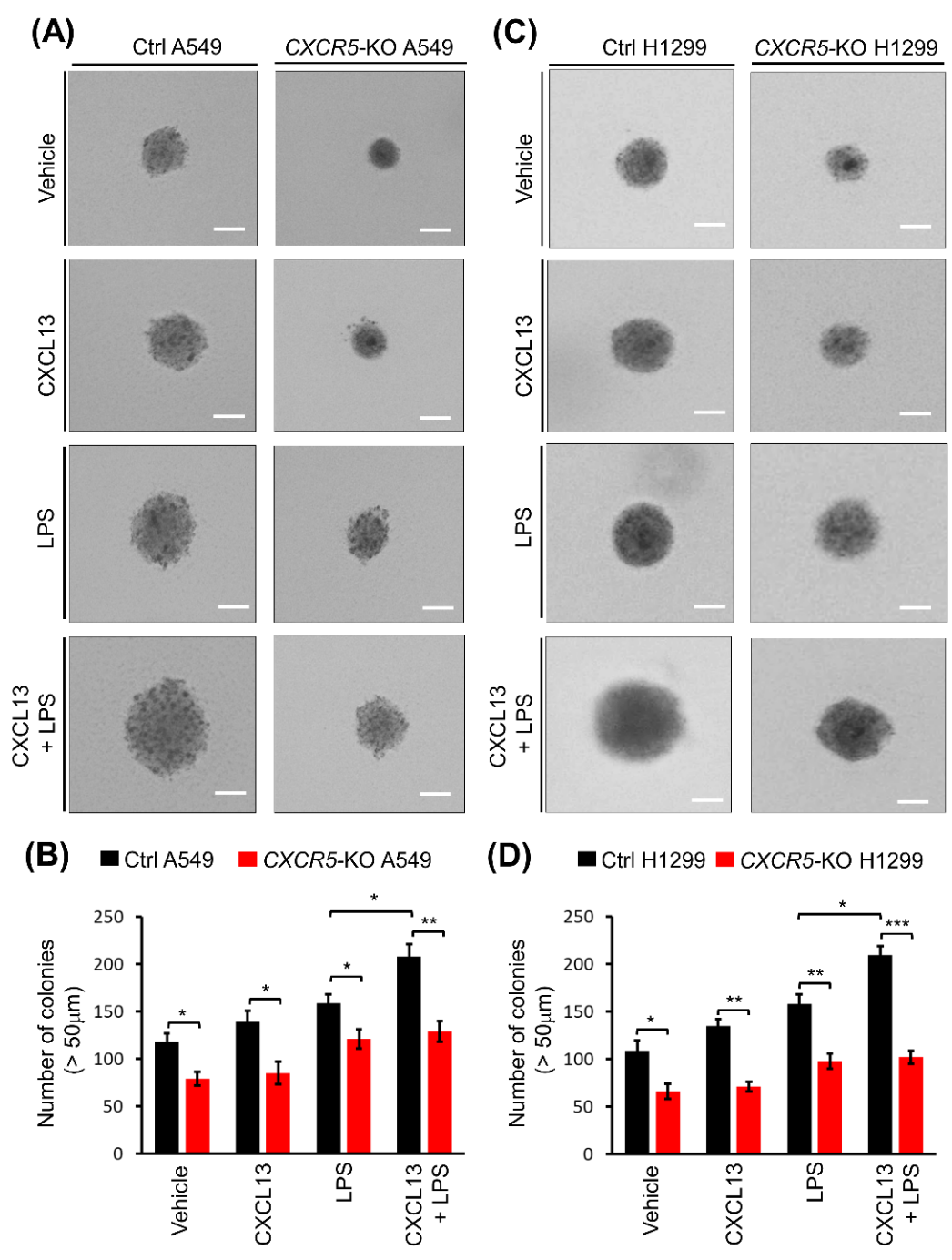


**Figure S9. Anchorage-independent colony formation assay in Ctrl A549 and *CXCR5*-KO A549 or Ctrl H1299 and *CXCR5*-KO H1299 in response to LPS, CXCL13, or co-treatment of LPS and CXCL13.** Ctrl A549 and *CXCR5*-KO A549 cells (**A** and **B**) or Ctrl H1299 and *CXCR5*-KO H1299 cells (**C** and **D**) were treated with vehicle (DMSO, 0.1% v/v concentration), LPS (10 μg/mL), CXCL13 (20 ng/mL), or co-treatment of LPS (10 μg/mL) and CXCL13 (20 ng/mL) for 24 days or 35 days, respectively. The number of colonies was counted. Results are presented as the mean ± SD of three independent experiments. Scale bar, 50 μm. * *P* < 0.05, ** *P* < 0.01, and *** *P* < 0.001.

**Table S1. Clinical Data of the Patients with Non-Small Cell Lung Cancer (NSCLC)**

| **Patient ID** | **Gender** | **Age (yr)** | **Histology (Cell type)** | **Stage** | **Surgery (Year)** | **Recurrence (Year)** | **Death (Year)** |
| --- | --- | --- | --- | --- | --- | --- | --- |
| **LTT01** | Man | 76 | Squamous cell carcinoma | Stage IIB | 2008 | 2009 | 2010 |
| **LTT02** | Man | 77 | Squamous cell carcinoma | Stage IIB | 2008 | . | 2008 |
| **LTT03** | Man | 75 | Others | Stage IIA | 2008 | 2014 | . |
| **LTT04** | Man | 65 | Others | Stage IIB | 2008 | 2009 | 2010 |
| **LTT05** | Man | 67 | Squamous cell carcinoma | Stage IIIA | 2009 | 2012 | 2014 |
| **LTT06** | Man | 67 | Squamous cell carcinoma | Stage 1B | 2009 | 2010 | 2011 |
| **LTT07** | Man | 64 | Others | Stage IIA | 2009 | 2010 | 2011 |
| **LTT08** | Man | 59 | Squamous cell carcinoma | Stage IIB | 2009 | 2011 | 2015 |
| **LTT10** | Woman | 65 | Adenocarcinoma | Stage 1B | 2009 | . | . |
| **LTT11** | Woman | 53 | Others | Stage IIA | 2010 | 2012 | 2014 |
| **LTT12** | Woman | 54 | Adenocarcinoma | Stage 1B | 2010 | . | . |
| **LTT13** | Woman | 56 | Adenocarcinoma | Stage 1B | 2010 | 2015 | . |
| **LTT14** | Man | 70 | Adenocarcinoma | Stage 1A | 2010 | . | . |
| **LTT17** | Woman | 68 | Adenocarcinoma | Stage 1B | 2010 | . | . |
| **LTT18** | Woman | 72 | Others | Stage 1B | 2010 | 2010 | 2013 |
| **LTT19** | Man | 69 | Others | Stage 1A | 2010 | . | . |
| **LTT20** | Man | 69 | Adenocarcinoma | Stage 1A | 2010 | . | . |
| **LTT21** | Woman | 60 | Adenocarcinoma | Stage 1B | 2010 | . | . |
| **LTT22** | Man | 84 | Adenocarcinoma | Stage 1B | 2010 | 2011 | 2012 |
| **LTT24** | Man | 70 | Adenocarcinoma | Stage IIB | 2010 | 2010 | 2016 |
| **LTT25** | Man | 59 | Adenocarcinoma | Stage 1B | 2010 | . | . |
| **LTT26** | Woman | 38 | Adenocarcinoma | Stage IIA | 2010 | . | . |
| **LTT27** | Man | 77 | Adenocarcinoma | Stage 1A | 2010 | . | 2015 |
| **LTT28** | Woman | 79 | Adenocarcinoma | Stage 1B | 2010 | 2011 | 2012 |
| **LTT29** | Woman | 47 | Adenocarcinoma | Stage 1B | 2010 | 2011 | 2015 |
| **LTT30** | Man | 81 | Adenocarcinoma | Stage 1B | 2010 | . | 2010 |
| **LTT32** | Woman | 66 | Adenocarcinoma | Stage IIA | 2010 | 2010 | 2013 |
| **LTT33** | Man | 75 | Adenocarcinoma | Stage 1A | 2012 | 2010 | 2016 |
| **LTT34** | Man | 65 | Adenocarcinoma | Stage 1A | 2010 | . | . |
| **LTT35** | Woman | 50 | Adenocarcinoma | Stage IIA | 2010 | . | . |
| **LTT36** | Man | 70 | Adenocarcinoma | Stage 1B | 2010 | . | . |
| **LTT38** | Man | 72 | Others | Stage IIA | 2010 | 2014 | 2016 |
| **LTT39** | Man | 68 | Adenocarcinoma | Stage 1B | 2010 | . | 2011 |
| **LTT42** | Woman | 59 | Adenocarcinoma | Stage IIA | 2010 | 2013 | 2015 |
| **LTT43** | Man | 70 | Adenocarcinoma | Stage 1B | 2010 | . | . |
| **LTT47** | Man | 63 | Others | Stage 1B | 2011 | . | . |
| **LTT48** | Man | 68 | Adenocarcinoma | Stage IIB | 2011 | . | 2012 |
| **LTT49** | Man | 67 | Squamous cell carcinoma | Stage IIB | 2011 | 2011 | 2014 |
| **LTT50** | Man | 72 | Squamous cell carcinoma | Stage 1B | 2011 | . | 2012 |
| **LTT51** | Woman | 73 | Adenocarcinoma | Stage 1B | 2011 | . | . |
| **LTT52** | Woman | 43 | Adenocarcinoma | Stage 1B | 2011 | . | . |
| **LTT53** | Man | 64 | Others | Stage 1B | 2011 | . | 2011 |

| **Table S2. Magnitude difference of ∆CXCR5, ∆CXCL13, and ∆TLR4 between lung tumor tissues (LTTs) and matched lung normal** | | | | | | | | | | |
| --- | --- | --- | --- | --- | --- | --- | --- | --- | --- | --- |
| **tissues (mLNTs)** | | | | | | | | | | |
| **Lung Tumor Tissues (LTTs)** | | | | **matched Lung Normal Tissues (mLNTs)** | | | | **∆Mag = LTT Mag – mLNT Mag** | | |
| LTTs (Patient's ID) | CXCR5 | CXCL13 | TLR4 | mLTTs (Patient's ID) | CXCR5 | CXCL13 | TLR4 | CXCR5 | CXCL13 | TLR4 |
| LTT20.AVG_Signal | 150.5316 | 1255.698 | 83.2689 | LNT20.AVG_Signal | 3.088452 | 10.54921 | 25.63334 | 147.443148 | 1245.14879 | 57.63556 |
| LTT01.AVG_Signal | 150.1103 | 753.0461 | 90.58291 | LNT01.AVG_Signal | 12.59483 | 14.56214 | 33.0638 | 137.51547 | 738.48396 | 57.51911 |
| LTT35.AVG_Signal | 112.5893 | 616.873 | 49.97831 | LNT35.AVG_Signal | -1.842923 | 14.20705 | 93.39867 | 114.432223 | 602.66595 | -43.42036 |
| LTT33.AVG_Signal | 114.7718 | 725.9642 | 12.03174 | LNT33.AVG_Signal | 6.078507 | 1.461449 | 38.81673 | 108.693293 | 724.502751 | -26.78499 |
| LTT05.AVG_Signal | 109.3759 | 343.0872 | 113.4038 | LNT05.AVG_Signal | 28.55904 | 163.8298 | 150.6057 | 80.81686 | 179.2574 | -37.2019 |
| LTT03.AVG_Signal | 78.77646 | 851.8094 | 116.4391 | LNT03.AVG_Signal | 6.0963 | 24.88099 | 118.8646 | 72.68016 | 826.92841 | -2.4255 |
| LTT12.AVG_Signal | 57.90016 | 67.0653 | 3.645279 | LNT12.AVG_Signal | -1.997747 | 3.755961 | 175.569 | 59.897907 | 63.309339 | -171.923721 |
| LTT14.AVG_Signal | 60.79114 | 630.2831 | 184.3722 | LNT14.AVG_Signal | 8.528769 | 25.96509 | 32.20562 | 52.262371 | 604.31801 | 152.16658 |
| LTT36.AVG_Signal | 45.14469 | 190.9306 | 7.798307 | LNT36.AVG_Signal | 6.587297 | 17.85246 | 131.1747 | 38.557393 | 173.07814 | -123.376393 |
| LTT38.AVG_Signal | 41.78409 | 344.2534 | 48.24511 | LNT38.AVG_Signal | 9.943066 | 23.90461 | 54.68033 | 31.841024 | 320.34879 | -6.43522 |
| LTT25.AVG_Signal | 33.05542 | 172.7861 | 75.29714 | LNT25.AVG_Signal | 3.594219 | -3.468579 | 50.21067 | 29.461201 | 176.254679 | 25.08647 |
| LTT32.AVG_Signal | 15.67215 | 33.10017 | 33.83539 | LNT32.AVG_Signal | -7.226534 | 5.176767 | 52.3441 | 22.898684 | 27.923403 | -18.50871 |
| LTT51.AVG_Signal | 29.0585 | 68.55112 | 2.725402 | LNT51.AVG_Signal | 9.4942 | 12.62507 | 60.35207 | 19.5643 | 55.92605 | -57.626668 |
| LTT50.AVG_Signal | 21.97272 | 61.12313 | 9.994733 | LNT50.AVG_Signal | 2.98225 | 5.785671 | 97.82095 | 18.99047 | 55.337459 | -87.826217 |
| LTT43.AVG_Signal | 38.45738 | 94.22348 | 11.18273 | LNT43.AVG_Signal | 19.57616 | 88.52506 | 128.2398 | 18.88122 | 5.69842 | -117.05707 |
| LTT17.AVG_Signal | -0.7712352 | 8.283772 | 22.72072 | LNT17.AVG_Signal | -13.91291 | 19.25745 | 47.64342 | 13.1416748 | -10.973678 | -24.9227 |
| LTT24.AVG_Signal | 56.95601 | 319.9933 | 45.74575 | LNT24.AVG_Signal | 44.33034 | 204.4035 | 105.5657 | 12.62567 | 115.5898 | -59.81995 |
| LTT07.AVG_Signal | 8.667205 | 136.6829 | 75.70138 | LNT07.AVG_Signal | -2.062512 | 12.05156 | 30.03092 | 10.729717 | 124.63134 | 45.67046 |
| LTT47.AVG_Signal | 14.94163 | 257.8791 | 6.811279 | LNT47.AVG_Signal | 4.247125 | 17.47937 | 88.91879 | 10.694505 | 240.39973 | -82.107511 |
| LTT27.AVG_Signal | 67.99383 | 457.8886 | 81.18542 | LNT27.AVG_Signal | 57.41436 | 139.838 | 115.8488 | 10.57947 | 318.0506 | -34.66338 |
| LTT42.AVG_Signal | 9.814507 | 20.58144 | 1.090492 | LNT42.AVG_Signal | -0.6627484 | 11.75689 | 26.55777 | 10.4772554 | 8.82455 | -25.467278 |
| LTT11.AVG_Signal | 17.15844 | 76.11288 | 5.051239 | LNT11.AVG_Signal | 7.088042 | 17.31109 | 71.8132 | 10.070398 | 58.80179 | -66.761961 |
| LTT08.AVG_Signal | 16.72429 | 114.1804 | -6.68653 | LNT08.AVG_Signal | 7.40232 | 8.987511 | 33.21156 | 9.32197 | 105.192889 | -39.89809 |
| LTT53.AVG_Signal | 25.2254 | 105.2338 | 44.8012 | LNT53.AVG_Signal | 17.72565 | 22.10356 | 48.48206 | 7.49975 | 83.13024 | -3.68086 |
| LTT26.AVG_Signal | -0.6724991 | -5.43645 | -5.623194 | LNT26.AVG_Signal | -2.444382 | 23.57616 | 29.04507 | 1.7718829 | -29.01261 | -34.668264 |
| LTT06.AVG_Signal | 3.998403 | 206.53 | 22.25689 | LNT06.AVG_Signal | 3.787045 | 20.35299 | 42.98742 | 0.211358 | 186.17701 | -20.73053 |
| LTT48.AVG_Signal | 6.591616 | 41.71946 | 11.30269 | LNT48.AVG_Signal | 8.675296 | 7.897731 | 64.8029 | -2.08368 | 33.821729 | -53.50021 |
| LTT30.AVG_Signal | 5.250968 | 191.7024 | 56.54182 | LNT30.AVG_Signal | 8.027169 | 72.32077 | 93.29726 | -2.776201 | 119.38163 | -36.75544 |
| LTT34.AVG_Signal | 8.04914 | 54.76478 | 27.11695 | LNT34.AVG_Signal | 12.82899 | 34.99398 | 89.40562 | -4.77985 | 19.7708 | -62.28867 |
| LTT52.AVG_Signal | 4.86598 | 41.65642 | 20.3118 | LNT52.AVG_Signal | 11.70561 | 3.125313 | 39.68243 | -6.83963 | 38.531107 | -19.37063 |
| LTT21.AVG_Signal | 3.717854 | 88.25748 | 17.83532 | LNT21.AVG_Signal | 14.01256 | 185.1917 | 273.1962 | -10.294706 | -96.93422 | -255.36088 |
| LTT19.AVG_Signal | 6.205079 | 960.6846 | 78.16854 | LNT19.AVG_Signal | 16.58968 | 67.45895 | 150.7512 | -10.384601 | 893.22565 | -72.58266 |
| LTT28.AVG_Signal | 32.56716 | 85.16618 | 41.42778 | LNT28.AVG_Signal | 43.16818 | 96.43972 | 100.021 | -10.60102 | -11.27354 | -58.59322 |
| LTT10.AVG_Signal | 10.65095 | 53.33712 | 127.7456 | LNT10.AVG_Signal | 22.25127 | 121.067 | 398.2639 | -11.60032 | -67.72988 | -270.5183 |
| LTT29.AVG_Signal | 3.178041 | 20.59893 | -0.0732936 | LNT29.AVG_Signal | 24.37698 | 11.21952 | 55.61167 | -21.198939 | 9.37941 | -55.6849636 |
| LTT49.AVG_Signal | 5.973418 | 50.39322 | 31.42113 | LNT49.AVG_Signal | 28.49196 | 99.72263 | 128.1317 | -22.518542 | -49.32941 | -96.71057 |
| LTT22.AVG_Signal | 4.570197 | 44.43365 | 31.66134 | LNT22.AVG_Signal | 27.96853 | 3.535346 | 187.2014 | -23.398333 | 40.898304 | -155.54006 |
| LTT04.AVG_Signal | 14.50103 | 174.6113 | 99.41924 | LNT04.AVG_Signal | 40.03155 | 126.1058 | 100.5673 | -25.53052 | 48.5055 | -1.14806 |
| LTT13.AVG_Signal | -7.476935 | 57.08435 | 64.50797 | LNT13.AVG_Signal | 18.41299 | 6.231636 | 120.5104 | -25.889925 | 50.852714 | -56.00243 |
| LTT18.AVG_Signal | 33.75057 | 119.1939 | 65.33083 | LNT18.AVG_Signal | 72.64797 | 134.7256 | 147.3634 | -38.8974 | -15.5317 | -82.03257 |
| LTT02.AVG_Signal | 10.35308 | 144.9056 | 12.57382 | LNT02.AVG_Signal | 49.40938 | 218.9223 | 50.55797 | -39.0563 | -74.0167 | -37.98415 |
| LTT39.AVG_Signal | 8.06956 | 19.02313 | -12.03278 | LNT39.AVG_Signal | 403.3254 | 495.7097 | 55.79289 | -395.25584 | -476.68657 | -67.82567 |
